# Potent Cytotoxic Analogs of Amphidinolides from the Atlantic Octocoral *Stragulum bicolor*

**DOI:** 10.3390/md17010058

**Published:** 2019-01-16

**Authors:** Genoveffa Nuzzo, Bruno de Araújo Gomes, Carmela Gallo, Pietro Amodeo, Clementina Sansone, Otília D. L. Pessoa, Emiliano Manzo, Rosa Maria Vitale, Adrianna Ianora, Evelyne A. Santos, Leticia V. Costa-Lotufo, Angelo Fontana

**Affiliations:** 1 Institute of Biomolecular Chemistry, National Research Council (CNR), 80078 Pozzuoli, Naples, Italy; carmen.gallo@icb.cnr.it (C.G.); pamodeo@icb.cnr.it (P.A.); emanzo@icb.cnr.it (E.M.); rmvitale@icb.cnr.it (R.M.V.); 2 Departamento de Química Orgânica e Inorgânica, Universidade Federal do Ceará, Fortaleza 60020-181, CE, Brazil; brunoaraujogomes1986@yahoo.com.br (B.d.A.G.); otilialoiola@gmail.com (O.D.L.P.); 3 Stazione Zoologica Anton Dohrn, Villa Comunale, 80121 Napoli, Italy; clementina.sansone@szn.it (C.S.); ianora@szn.it (A.I.); 4 Departamento de Farmacologia, Instituto de Ciências Biomédicas, Universidade de São Paulo, São Paulo 11030-400, SP, Brazil; alvesevelyne@yahoo.com.br (E.A.S.); costalotufo@gmail.com (L.V.C.-L.)

**Keywords:** amphidinolides, *Stragulum bicolor*, cytotoxic macrolides, stereochemistry

## Abstract

Amphidinolides are cytotoxic macrolides produced by symbiotic unicellular microalgae of the genus *Amphidinium*. Here we describe the identification of four related molecules belonging to this macrolide family isolated from the invertebrate *Stragulum bicolor*. The new molecules, named amphidinolide PX1-PX3 and stragulin A (**1**–**4**), show an unprecedented carbon skeleton whose complete stereochemistry has been determined by spectroscopic and computational methods. Differences in the structures of these molecules modulate their biological activity in a panel of tumor cell lines, but the opened derivative stragulin (**4**) shows a very potent and specific cytotoxic activity (IC_50_ 0.18 µM) against the aggressive human melanoma cell A2058.

## 1. Introduction

**Scheme 1:**
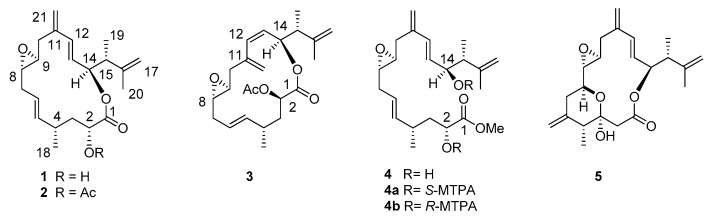
Amphidinolide PX1-PX3 (**1**–**3**), stragulin A (**4**) with related Mosher’s derivatives **4a** and **4b** and amphidinolide P (**5**).

Amphidinolides (AMPs) and related compounds are a diverse class of more than 40 macrolides with extremely high cytotoxicity against several carcinoma cell lines [1,2,3,4]. The most potent components of the family show a 26-membered macrocycle and are active at picogram level against murine lymphoma cells (L1210) and human epidermoid carcinoma cells (KB). The mechanisms of action have not been detailed for every member of the family but more than one AMPs disrupt microfilament organization of cytoskeleton [5,6,7,8]. It has also been proposed that small (15- and 16-membered) and large (26-membered) macrocycles target actin-G or actin-F alternatively. Together with similar macrolides reported with other names (amphidinolactones, iriomoteolides and caribenolide I) [9,10,11,12,13] and related linear polyketides (e.g., amphidinins) [14,15,16,17], AMPs are produced by dinoflagellates of the genus *Amphidinium*. Recently, we have reported the extraction of a number of amphidinolides from the softcoral *Stragulum bicolor* [18,19], including the known amphidinolide P and T1 and three new analogues named amphidinolide C4, B8 and B9 according to the structural relationship with other known members of the family. Here we describe the full characterization of three new cytotoxic members of the AMP family (**1**–**3**), together with the linear polyketide stragulin A (**4**) that showed cytotoxic activity at sub-micromolar scale against melanoma cells. Although the macrolides **1**–**3** show a new carbon skeleton, they are named amphidinolide PX1-PX3 for the partial analogy with the 15-membered amphidinolide P **5** (Scheme 1) [20].

## 2. Results and Discussion

Frozen specimens of *S. bicolor* were soaked in cold MeOH and extracted in five fractions (A-E) by our standard hydrophobic polystyrene-divinylbenzene resin (HR-X) for solid phase extraction (SPE) protocol [21]. After HPLC purification, fraction B yielded the new 15-membered macrolide amphidinolide PX1 (AMP-PX1, **1**) and stragulin A (**4**) together with amphidinolide B8, B9 and C4, whereas the new amphidinolide PX2 (AMP-PX2, **2**) and amphidinolide PX3 (AMP-PX3, **3**) were obtained from the SPE fraction C along with the known amphidinolide P and T1.

**Figure 1:**
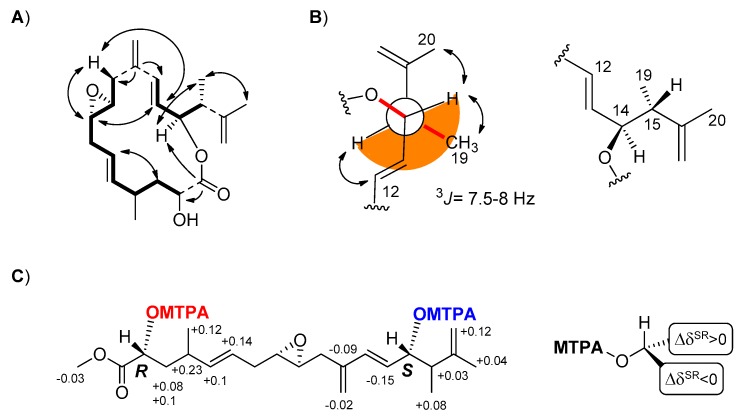
Stereochemical assignment of compounds **1**–**4**. (**A**) Key correlations to support the relative stereochemistry of **1** and **2**. Double arrow = Nuclear Overhauser Effect (NOE); simple arrow = Heteronuclear Multiple Bond Coherence (HMBC); (**B**) Newman projection of the C-12/C-20 fragment of amphidinolide PX1 (**1**) and PX2 (**2**) with the *anti* configuration of the substituents at C-14 and C-15. Double arrow = NOE; (**C**) Chemical shift differences Δδ_(δSester−δRester)_ between (*S*)- and (*R*)-α-Methoxy-α-(trifluoromethyl)phenylacetic acid (MTPA) derivatives of stragulin A (**4**).

Based on the sodium adduct at *m*/*z* 369.2052 [M+Na]^+^ detected in high-resolution electrospray ionization mass spectrometry (HR ESIMS), the molecular formula C_21_H_30_O_4_ was established for AMP-PX1 (**1**). Analysis of the NMR data (Supplementary Material, Tables S1 and S2, Figures S1–S5) revealed the presence of one carbonyl ester function and four double bonds including two exomethylene groups, which justifies five of the seven degrees of unsaturation predicted by the molecular formula. The last two unsaturations were associated with an epoxide cycle (δ_H-8_ = 2.63, δ_C-8_ = 59.3; δ_H-9_ = 2.53, δ_C-9_ = 57.0) and to a macrolide ring according to the deshielded NMR chemical shift of the carbinol proton at 5.37 ppm (δ_C-14_ = 77.8). The molecule also contained six methine (four of them bearing oxygen atoms) and three methyl groups whose connectivities were fully resolved by Correlation Spectroscopy (COSY) and Total Correlation Spectroscopy (TOCSY) spectra in two distinct spin-systems from C-2 to C-10 and from C-12 to the methyl group on C-15 (Figure 1A). The ^l^H-^13^C long-range correlations of the sp^2^ carbon at 141.2 ppm (C-11) with both the methylene protons at δ 2.64-2.07 (H_2_-10) and the olefinic signal at δ 6.28 (H-12) allowed us to join the two spin systems through the exomethylene bridge at C-11. Another diagnostic long-range cross-peak linked the carbinol proton at δ 5.37 with the carbonyl carbon at δ 174.6, thus closing the macrolide ring. The remaining carbons and protons were all inferred from the clear correlations with the above signals in agreement with the planar structure of **1**.

Amphidinolide PX2 (AMP-PX2, **2**) showed a sodium adduct ion [M+Na]^+^ at *m*/*z* 411.2146 for the molecular formula C_23_H_32_O_5_ and a ^1^H NMR spectrum with signals similar to those of **1**. The two compounds mostly differed for the downfield shift of the carbinol proton H-2 at δ 5.04 and the presence of an acetyl group in AMP-PX2 (**2**). In addition to minor changes in the signals of the spatially neighboring protons at C-3, C-13, C-15 and C-17 (Supplementary Material, Tables S1 and S2, Figures S1–S16), the acetylation at C-2 was diagnostically supported by correlation of the carbonyl at 169.7 ppm with both oxymethine protons at δ 5.04 (H-2) and 5.33 (H-14), whereas the acetyl carbonyl moiety at δ 170.0 showed cross-peaks only with the methyl group at δ 1.73 and the carbinol signal at δ 5.04 (H-2). In **1** and **2**, the *E* geometry of the two vicinally di-substituted double bonds was deduced from the *J* values above 15.5 Hz of the olefinic proton pairs H5/H6 and H12/H13, whereas the small coupling constant of 2.0 Hz between H-8 and H-9, as measured by homo-decoupling experiments on **2**, suggested a *trans* configuration of the epoxide ring. The relative stereochemistry of AMP-PX2 (**2**) was completed by two-dimensional Nuclear Overhauser Effect Spectroscopy (2D-NOESY) experiments that confirmed the depicted structure [20]. The ^3^*J* coupling constant of 7.5-8 Hz between H-14 and H-15 suggested a dihedral angle of 160° in agreement with the *anti* configuration of the substituents at C-14 and C15 shown for both **1** and **2** in Figure 1B. This assignment was corroborated by the correspondence of the chemical shift of H-14 and Me-19 with those previously reported for amphidinolide P (**5**) and its 3-O-methyl derivative [18,20,22,23].

Mass spectrum of AMP-PX3 (**3**) displayed a sodium adduct at *m*/*z* 411.2140 identical to compound **2** (C_23_H_32_O_5_Na^+^), but a ^1^H NMR spectrum with significant differences from that of AMP-PX2 (**2**) (Supplementary Material, Tables S1–S3). A closer inspection of the NMR data of **3** in C_6_D_6_ or CDCl_3_ (Supplementary Material, Figure S17–S25) revealed an upfield shift and a change in ^3^*J* value of the olefin protons H-12 (5.73, d, *J* = 11.5 Hz) and H-13 (5.38, dd, *J* = 11.5, 10.0) suggesting a *Z* configuration of the double bond. Analysis of the 2D-NMR spectra supported this hypothesis and showed an upfield shift of C-11 and C-14 in agreement with the *Z* stereochemistry of the C-12/C-13 double bond in **3**. It is worth noting that this is the first finding of *Z* double bonds in the congested 15-membered macrocycle of amphidinolides [3,4]. The remaining signals were in agreement with the depicted structure of **3** and, despite the differences in the NMR chemical shifts, supported a structure with the same stereochemistry and substitution pattern of **2**.

The last compound isolated from *S. bicolor* was identified as stragulin A (**4**) whose ^1^H-NMR spectrum did not contain the typical downfield shift of the oxymethine signal H-14 due to the macrolide closure. Accordingly, compound **4** showed a HR ESIMS molecular ion at *m*/*z* 401.2315 [M+Na]^+^ in agreement with the molecular formula C_22_H_34_O_5_ and only six degrees of unsaturation. The ^1^H NMR spectrum of **4** was similar to that of AMP-PX1 (**1**) and 2D NMR experiments (Supplementary Material, Figure S26–S31) allowed us to build the same spin systems of the macrolides **1**–**3**. Together with the shift of the carbinol group (H-14) from δ 5.37 in **1** (H-14) to δ 3.86 in **4**, this latter compound showed an additional methoxy group (δ_H_ 3.26, s). On the whole, these data indicated a linear structure with a carbon skeleton formally derived by the opening of the macrocycle ring of **1**. At the moment we cannot establish whether the occurence of this product is due to enzymatic reaction during the biosynthesis or to a spontaneous methanolysis of the closed analogs (e.g., **1** and **2**) during the chemical work-up.

The presence of two free secondary alcohols in **4** gave us the opportunity to establish the absolute stereochemistry of the molecule. From 200 µg of stragulin A (**4**) we obtained the diastereomeric Mosher’s derivatives **4a** and **4b** that were fully characterized by 2D NMR experiments (Supplementary Material, Figures S32–S37). Analysis of these data supported Δδ*^S,R^* values in full agreement with 2*R* and 14*S* configuration of **4** (Figure 1C). Since stragulin A (**4**) shares the same carbon skeleton of the macrolides **1**–**3**, this assignment also established the configuration of **1**–**3** as 2*R*, 8*S*, 9*S*, 14*S*, 15*R*. The stereochemistry of C-14 is in agreement with the suggested absolute configuration of amphidinolide P (**5**) isolated by Kobayashi [20], thus clarifying a conundrum deriving from the synthesis of this latter compound [24].

**Figure 2:**
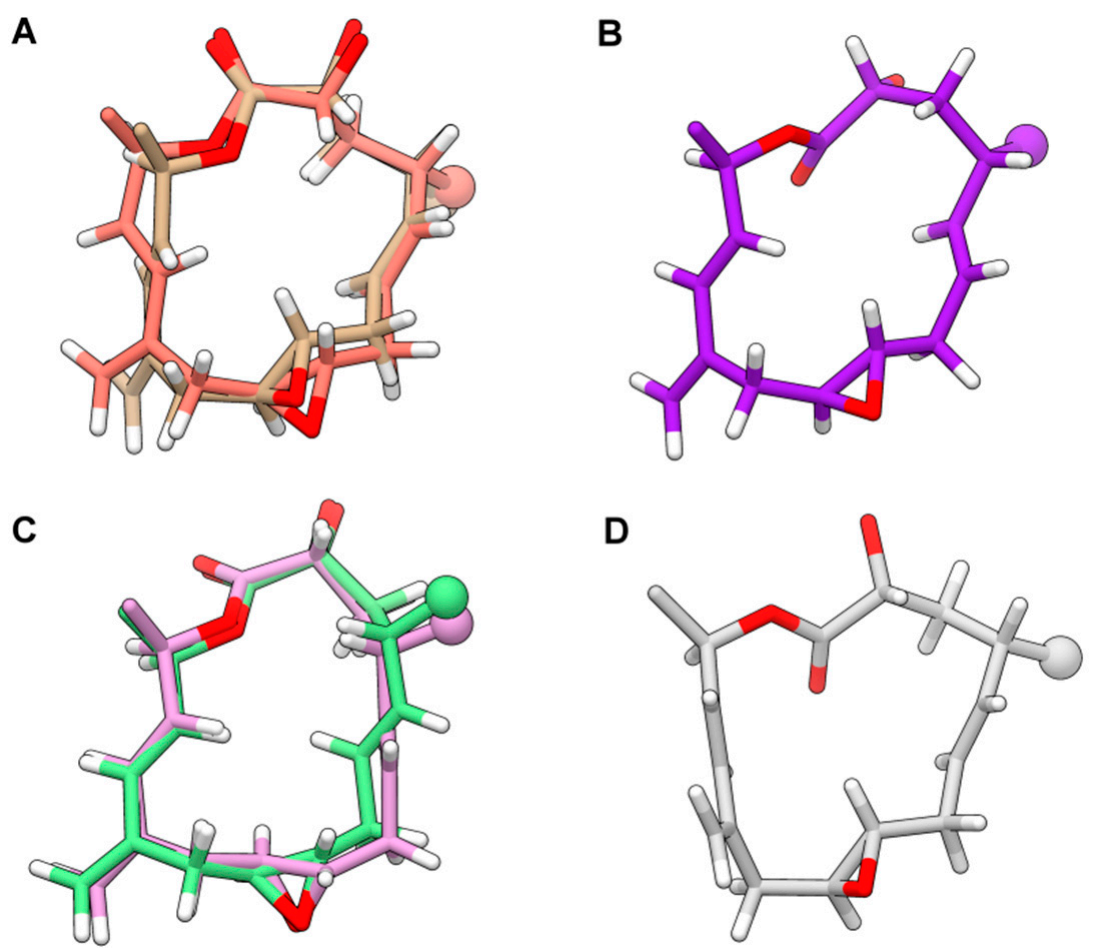
Restrained Energy Minimization (rEM) structures of amphidinolide PX2 (**2**) representative conformers. Panel (**A**): conformers I (beige) and V (salmon); panel (**B**): conformer II (violet); panel (**C**): conformers III (pink) and IV (light green); panel (**D**): conformer VI (gray). Structures are best-fit of the macrocycle atoms. Oxygen and hydrogen atoms are colored red and white, respectively. Methyl groups are shown as spheres colored as the carbon atoms of the corresponding conformer (colors in parenthesis).

The absolute stereochemistry at C4 of **1**–**3** could not be determined by chemical or direct spectroscopic methods; thus we performed a rigorous conformational study of AMP-PX2 (**2**) supported by experimental distance restraints (Supplementary Material, Table S4) derived from 2D-NOESY spectra in C_6_D_6_ and CDCl_3_ (Supplementary Material, Tables S1–S3, Figure S38) [25,26,27]. Since preliminary calculations showed the occurrence of conformational equilibria in solution, an iterative computational protocol based on repeated cycles of Simulated Annealing (SA)/Molecular Dynamics (MD)/Energy Minimization (EM) was applied to both possible epimers at C-4 of **2** (Supplementary Material, Tables S4–S6). The analysis of these results led to ruling out the 4*R* epimer on the basis of the significant violations in H-2/H-4 and H-2/Me-18 distance restraints, which were reversed in comparison with those calculated from NMR data. On the other hand, the ensemble of conformers with minimal sum of violations for the final pool of 25 conformers for the 4*S* epimer showed a convergence to six conformations of the 15-membered ring. Albeit limited, the flexibility of the 1,2-dimethyl-prop-2-en-1-yl and acetyl groups excessively increased the number of total conformers to be included in the final ensemble generation stage. For this reason, after verifying the consistency between experimental NOE patterns involving protons in the 1,2-dimethyl-prop-2-en-1-yl group and the corresponding possible distances in selected conformers, we performed the final analysis of violations only on the macrocyclic core together with the groups rigidly-anchored. The resulting six conformers shown in Figure 2 exhibited no significant violations on the whole set of 93 NOE- and antiNOE-derived restrains composed of (Supplementary Material, Table S5). These structures accounted for the flexibility due to: (i) different conformations of the lactone group; (ii) the *t-g+/g+-g-/g-t* conformations of the C2-C3 and C3-C4 bonds; (iii), the *syn*(H4-H5) or *syn*(H4-H6) orientation (with respect to the average plane of the macrocycle), due to the “flipping” of the C5-C6 endocyclic double bond; (iv) the *s-trans* or *s-cis* conformation of the C13-C12-C11-C21 conjugate system; and (v) the *syn*(H4-C19) or *anti*(H4-C19) orientation of the exocyclic C21 methylene group in the *s-cis* conformation. Among the six conformers, structures differed only in the *s-cis* vs. *s-trans* conformation of the conjugate system and the relative orientation of the H4-H5/H4-H6 proton pairs (Figure 2A,C, respectively).

The assignment of the *S* configuration at C-4 of AMP-PX2 (**2**) completed the stereochemical characterization of this product. Although we have not correlated by chemical methods the stereochemistry of **4** to **1**–**3**, we suggest that the 4*S* configuration is shared by the other products of *S. bicolor* because of a common biogenesis of the polyketide chain. This leads us to propose the 2*R*, 4*S*, 8*S*, 9*S*, 14*S*, 15*R* configuration of the six stereocenters in AMP-PX **1**–**3** and stragulin A (**4**). Together with amphidinolide Q [28], the macrocycle of amphidinolides PX1-3 (**1**–**3**) is the smallest carbon skeleton so far reported in the amphidinolide family.

In order to underline differences in the biological response of the four natural products, compounds **1**–**4** were tested between 8 µM to 8 nM against the melanoma A2058 cells derived from metastatic site (lymphonode), the human ovarian adenocarcinoma A2780 and the human neuroblastoma SK-N-BE-2 cell lines (Table 1). The linear polyketide **4** was strongly and selectively active on the highly invasive melanoma cell lines A2058 with an IC_50_ of 0.18 µM after 48 h of treatment. The related macrolide **1** was not active on the same cancer cell line but exhibited potent cytotoxicity against human ovarian adenocarcinoma with an IC_50_ of 4.3 µM after 48 h from the treatment (Table 1). Interestingly both acetylated derivatives **2** and **3** were inactive in the range of tested concentrations on these cell lines, as well as inactive up to 30 µM against a panel of human cell lines composed of colon adenocarcinoma HT-29, breast adenocarcinoma MCF-7 and melanoma SK-Mel-19 (Supplementary Material, Table S7).

## 3. Conclusions

Soft corals are known to produce a wide array of secondary metabolites but compounds **1**–**4** of *S. bicolor* are probably derived by a symbiotic dinoflagellate that is hosted by the invertebrate. This is inferred by the similarity of **1**–**3** with amphidinolide P (**5**) and by the co-occurrence of other known amphidinolides in the organic extract of the soft coral. In line with previous studies, the activity of these compounds on tumor cell lines suggests a clear dependence on tight structural determinants since a simple acetylation at C-2 or a modification of the stereochemistry of the double bond C-12/C-13 can result in the complete loss of activity. Although the specific cytotoxicity of stragulin A (**4**) on melanoma A2058 cells is interesting in relation to the dysregulation of apoptosis genes that govern survival of this tumor cell line, the significance of this result is linked to the selective activity in comparison to the absence of response elicited by the other related compounds.

We did not carry out any biosynthetic study on **1**–**4** in *S. bicolor*. However, their biogenesis could be related to that of amphidinolide P from *Amphidinium* sp., as described by the group of Prof. Kobayashi [15].

## 4. Materials and Methods

### 4.1. General

Optical rotations were measured on a Jasco P2000 digital polarimeter. UV spectra were acquired by a Jasco V-650 Spectrophotometer and Infrared (IR) spectra by a Jasco Fourier-transform (FT)-IR 4100 spectrophotometer (JASCO EUROPE s.r.l., Cremella (Lc), Italy). NMR spectra were recorded on Bruker DRX 600 spectrometer (600 MHZ for ^1^H, 150 MHz for ^13^C) equipped with a three channel inverse (TCI) CryoProbe. Chemical shifts values are reported in ppm (δ) and referenced to internal signals of residual protons (C_6_D_6_^1^H δ 7.15, ^13^C 128.0 ppm; CDCl_3_^1^H δ 7.26, ^13^C 77.0 ppm). High resolution mass spectra were acquired on a Q-Exactive Hybrid Quadrupole-Orbitrap Mass Spectrometer (Thermo Scientific, Milan, Italy). HPLC analyses were performed on a Jasco system (PU-2089 Plus-Quaternary Gradient Pump equipped with a Jasco MD-2018 Plus Photodiode Array Detector (Jasco, Cremella, Italy). Solid phase extraction (SPE) was carried out using polystyrene-divinyl benzene resin (CHROMABOND HR-X, Macherey-Nagel, Düren). Silica gel chromatography was performed using precoated Merck F254 plates. All the chemicals and solvents (Sigma-Aldrich, St. Louis, MO, USA) were of analytical reagent grade and were used without any further purification.

### 4.2. Sample Collection

*Stragulum bicolor* was collected on the underside of beach rocks in the intertidal zone along Caponga beach (04°02′ S 38°11′ W) located 61 Km east from Fortaleza, Ceará, Brazil. To prevent contamination, the animal was collected using clamps and metal spatulas, washed with sea water at the collection point and with deionized water in the laboratory. Specimen identification was conducted by Prof. Tito Monteiro da Cruz Lotufo at University of São Paulo, and a voucher sample was deposited at the Museum of Zoology of the University of São Paulo.

### 4.3. Cell Cultures and Viability Assay

Human neuroblast SK-N-BE (ATCC CCL185) were cultured in Eagle’s minimum essential medium (EMEM) supplemented with F12 Medium (1:1, *v*/*v*), 10% (*v*/*v*) fetal bovine serum (FBS) and 100 units mL^−1^ penicillin and 100 µg mL^−1^ streptomycin. The A2780 human ovarian cancer cell line (93112519 SIGMA) were cultured in RPMI-1640 medium supplemented with 2 mM Glutamine, 10% (*v*/*v*) fetal bovine serum (FBS) and 100 units mL^−1^ penicillin and 100 µg mL^−1^ streptomycin. The A2058 human melanoma cancer cell line (purchase from ATCC CRL-11147, Manassas, VA, USA) in Dulbecco’s modified Eagle’s medium (DMEM) supplemented with 10% (*v*/*v*) fetal bovine serum (FBS), 100 units mL^−1^ penicillin and 100 µg mL^−1^ streptomycin. All cell lines were incubated in a 5% CO_2_ humidified chamber at 37 °C for growth. SK-N-BE, A2780 and A2058 cells (20 × 10^3^ cells well^−1^) were seeded in a 96-well plate and kept overnight for attachment. The next day, the medium was replaced with fresh medium containing compounds. The concentrations tested for each compound were 0.003, 0.03, 0.3 and 3 µg mL^−1^. Cells were allowed to grow for 24 h and 48 h, and adherent cells were then examined for viability. Cells were incubated with 10 µL (10 µg mL^−1^) of MTT (3-[4,5-methylthiazol-2yl]-2,5-diphenyl-tetrazoliumbromide, Applichem A2231). After 3 h of incubation, the supernatant was removed and the resultant formazan crystals were dissolved in isopropyl alcohol (100 μL). Absorbance intensity was measured by a microplate reader (Multiskan FC, THERMO SCIENTIFIC), using a wavelength of 570 nm. All experiments were performed in triplicate, and the number of viable cells was calculated as the ratio between mean absorbance of sample and mean absorbance of untreated control cells and expressed as percentage viability.

Compounds **2** and **3** were also evaluated against human adenocarcinoma colon (HCT-116), breast (MCF-7) and melanoma (SK-Mel-19) cancer cell lines. Cells were maintained in DMEM medium supplemented with 10% fetal bovine serum (*v*/*v*) at 37 °C under a 5% CO_2_ atmosphere. For screening, cells were plated into 96-well plates (5 × 104 cells mL^−1^) and compounds were tested at concentrations ranging from 0.0001 to 13 µg/mL during 72 h of incubation. The effect on cell proliferation was determined by reduction of yellow dye 3-(4,5-dimethyl-2-thiazol)-2,5-diphenyl-2H-tetrazolium bromide (MTT) to a blue formazan product described by Mosmann, 1983. Doxorubicin was used as positive control. IC_50_ values along with 95% confidence intervals were calculated by nonlinear regression using GraphPad Prism 5.0 (Intuitive Software for Science).

### 4.4. Extraction and Isolation of Metabolites

The octocoral colonies (dry weight after extraction 4.1 g) were successively extracted with MeOH (3 × 300 mL). This dry extract (700 mg) was resuspended in 2 mL water and fractionated on 6.5 g HR-X resin. As reported in the previous work [19] preliminary LC-MS and NMR analyses of CH_3_CN/H_2_O (7:3) fraction (18.2 mg) and CH_3_CN (11.9, mg) showed the presence of amphidinolide derivatives and other minor related compounds. HPLC purification of the first fractions on C18 column with a gradient of CH_3_CN/H_2_O led to the isolation of amphidinolides B8, B9 and C4, together with new compounds stragulin A (**4**, 0.3 mg) and amphidinolide PX1 (**1**, 0.1 mg); whereas purification of the second fraction gave, along with the known amphidinolide P and T1, the new amphidinolides PX2 (**2**, 0.3 mg) and PX3 (**3**, 0.3 mg).

**Amphidinolide-PX1 (AMP-PX1) (1).** Colourless oil (100 µg): [α]_25_^D^-17 (*c* 0.006, CH_2_Cl_2_); UV. *λmax* (log ε) 203 nm (3.04), 231 nm (ε 3.49) IR (KBr) *νmax* 3394, 2922, 1730, 1376, 1200 cm^−1^; HR-ESIMS [M+Na]^+^ *m*/*z* 369.2052 (calcd. for C_21_H_30_NaO_4_^+^ 369.2036); ^1^H NMR and ^13^C NMR data, see Tables S1 and S2 (Supplementary Material).

**Amphidinolide-PX2 (AMP-PX2) (2).** Colourless oil (300 µg): [α] _25_^D^ +90 (*c* 0.02, CH_2_Cl_2_); UV. *λmax* (log ε) 203 nm (3.09), 231 nm (3.50), IR (KBr) *νmax* 2930, 1745, 1455, 1372, 1236 cm^−1^; HR-ESIMS [M+Na]^+^ *m*/*z* 411.2146 (calcd. for C_23_H_32_NaO_5_^+^ 411.2142); for ^1^H NMR and ^13^C NMR data, see Tables S1–S3 (Supplementary Material).

**Amphidinolide-PX3 (AMP-PX3) (3).** Colourless oil (300 µg): [α] _25_^D^ +19 (*c* 0.02, CH_2_Cl_2_); UV. *λmax* (log ε) 203 nm (3.10), 231 nm (3.50), IR (KBr) *νmax* 2931, 1748, 1447, 1368, 1226 cm^−1^; HR-ESIMS [M+Na]^+^ *m*/*z* 411.2140 (calcd. for C_23_H_32_NaO_5_^+^ 411.2142); ^1^H NMR and ^13^C NMR data, see Tables S1–S3 (Supplementary Material).

**Stragulin A (4)**. Colourless oil (300 µg): [α] _25_^D^ +50 (*c* 0.02, CH_2_Cl_2_); UV *λmax* (log ε) 203 nm (3.08), 231 nm (3.51), IR (KBr) *νmax* 3392, 2923, 1728, 1366, 1210 cm^−1^; HR-ESIMS [M+Na]^+^ *m*/*z* 401.2315 (calcd. for C_22_H_34_NaO_5_^+^ 401.2298); ^1^H NMR and ^13^C NMR data, see Tables S1 and S2 (Supplementary Material).

### 4.5. Preparation of MTPA Esters of Stragulin A (***4***)

(*R*)- and (*S*)-MTPA-Cl (10 μL) and a catalytic amount of 4-dimethylaminopyridine (DMAP) were separately added to two sample of **4** (0.1 mg each) in dry CH_2_Cl_2_ (0.5 mL). The resulting mixtures were allowed to stand at room temperature for 12 h. After the evaporation of the solvent, the mixtures were purified on RP-HPLC C18 using acetonitrile (ACN) as eluent, affording pure (*S*)- and (*R*)-MTPA esters **4a** and **4b**, respectively. (*S*)-MTPA ester of **4**: selected ^1^H NMR values (CDCl_3_, 600 MHz) 6.31 (1H, d, *J* = 15.0 Hz, H-12), 5.51 (1H, overlapped, H-13), 5.50 (1H, overlapped, H-14), 5.40 (1H, m, H-6), 5.34 (1H, overlapped, H-5), 5.20 (1H, overlapped, H-2), 5.18 (2H, s, H_2_-21), 4.82 (2H, s, H_2_-17), 3.74 (3H, s, OCH_3_), 2.55 (1H, m, H-15), 2.34 (1H, m, H-4), 1.96 (1H, m, H-3), 1.83 (1H, m, H-3), 1.69 (3H, s, H_3_-20), 1.06 (3H, s, H_3_-18), 1.03 (3H, s, H_3_-19); ESIMS *m*/*z* 833.3 [M+Na]^+^. (*R*)-MTPA ester of **4**: selected ^1^H NMR values (CDCl_3_, 600 MHz) 6.40 (1H, d, *J* = 16.0 Hz, H-12), 5.66 (1H, overlapped, H-13), 5.53 (1H, overlapped, H-14), 5.26 (1H, m, H-6), 5.24 (3H, H-5 and H_2_-21), 5.15 (1H, dd, *J* = 11.0, 2.5 Hz H-2), 4.70 (2H, s, H_2_-17), 3.77 (3H, s, OCH_3_), 2.52 (1H, m, H-15), 2.11 (1H, m, H-4), 1.88 (1H, m, H-3), 1.73 (1H, m, H-3), 1.65 (3H, s, H_3_-20), 0.95 (3H, s, H_3_-19), 0.94 (3H, s, H_3_-18); ESIMS *m*/*z* 833.3 [M+Na]^+^.

### 4.6. Protocol and Results for the Determination of Molecular 3D Structure and Absolute Configuration at C4 Stereocenter Of Compound (***2***)

NOE effects from 2D-NOESY spectra were classified into either unambiguous (uNOEs, not involving methylene protons requiring stereochemical assignment), or initially-ambiguous (iaNOEs, involving methylene protons to be stereochemically assigned), or “surely-missing” (antiNOEs) and then translated into distance restraints. Repeated cycles (n = 10) of Simulated Annealing (SA) and Molecular Dynamics (MD) were run for both possible epimers at C-4. Since preliminary calculations showed the occurrence of conformational equilibria in solution, preventing the simultaneous satisfaction of the whole restraint set by a single conformation, the final calculations were run with the stereochemically-unambiguous antiNOE restraints alone, that is, 105 inter-proton distances above 3.2 Å (3.7 Å for restraints including methyl groups, see Supplementary Material, Table S4). Structures from all MD trajectories were clustered on heavy atom root-mean-square deviation (RMSD) for 4*S* and 4*R* epimers. The representative structures of the most populated clusters globally including >80% of the total sampled MD frame conformations for each epimer were refined by restrained Energy Minimization (rEM) with the same set of constraints employed in SA/MD cycles. The resulting conformers, after elimination of redundant structures, formed the “basic” conformer ensemble of the two epimers. Since all conformers for both epimers were consistent with the same stereochemical assignment of initially-ambiguous restraints, this latter was used for the subsequent selection stage. In particular, all restraints only involving protons attached to C-atoms belonging to the macrocycle or rigidly-anchored to it (i.e., 65 antiNOEs, with the corresponding lower limit distance increased by 0.3 Å, plus 28 NOEs providing 28 lower + 28 upper limits for each set of each diastereomer, Supplementary Material, Table S5), were used to select the minimal ensembles of conformers exhibiting the lowest sum of restraint. Each restraint systematically violated in the “basic” ensemble of each diastereomer was added (or enforced by increasing the corresponding lower limit by 0.3 Å in the case of antiNOEs) to the restraints used in SA, to perform a new set of 10 SA/MD cycles for the corresponding diastereomer, to correct possible undersampling or other biases in the SA/MD exploration. The resulting structures, after the same clustering, EM refinement (excluding the restraint added in the SA/EM stage) and selection performed on the “basic” ensembles, were added to the final pool of selected structures to be used in the final analyses.

## Figures and Tables

**Table 1:** Cytotoxicity (IC_50_) of AMP-PXs **1**–**3** and stragulin A (**4**) on tumor cell lines. Each compound was tested in triplicate. Cells were allowed to grow for 48 h and adherent cells were then examined for viability by 3-(4,5-dimethylthiazol-2-yl)-2,5-diphenyltetrazolium bromide tetrazolium reduction (MTT) assay.

Compound	Melanoma (A2058 Cell Line)	Ovarian Adenocarcinoma (A2780 Cell Line)	Neuroblastoma (SK-N-BE-2 Cell Line)
AMP-PX1 (**1**)	n.a.*	4.3 µM	n.a.
AMP-PX2 (**2**)	n.a.	n.a.	n.a.
AMP-PX3 (**3**)	n.a.	n.a.	n.a.
Stragulin A (**4**)	0.18 µM	n.a.	n.a.

* n.a. = not active.

## Supplementary Materials

The following are available online at http://www.mdpi.com/1660-3397/17/1/58/s1, NMR assignments, Tables S1–S7, NMR spectra Figures S1–S38, Refinement protocol of Simulated Annealing (SA), Molecular Dynamics (MD) and Energy Minimization (EM).

## References

[1] Kobayashi J., Kubota T. (2007). Bioactive Macrolides and Polyketides from Marine Dinoflagellates of the Genus *Amphidinium*. J. Nat. Prod..

[2] Kobayashi J., Ishibachi M. (1993). Bioactive Metabolites of Symbiotic Marine Microorganisms. Chem. Rev..

[3] Kobayashi J. (2008). Amphidinolides and its related macrolides from marine dinoflagellates. J. Antibiot..

[4] Kobayashi J., Tsuda M. (2004). Amphidinolides, bioactive macrolides from symbiotic marine dinoflagellates. Nat. Prod. Rep..

[5] Saito S.Y., Feng J., Kira A., Kobayashi J., Ohizumi Y. (2004). Amphidinolide H, a novel type of actin-stabilizing agent isolated from dinoflagellate. Biochem. Biophys. Res. Commun..

[6] Usui T., Kazami S., Dohmae N., Mashimo Y., Kondo H., Tsuda M., Terasaki A.G., Ohashi K., Kobayashi J., Osada H. (2004). Amphidinolide H, a Potent Cytotoxic Macrolide, Covalently Binds on Actin Subdomain 4 and Stabilizes Actin Filament. Chem. Biol..

[7] Sánchez D., Andreou T., Costa A.M., Meyer K.G., Williams D.R., Barasoain I., Díaz J.F., Lucena-Agell D., Vilarrasa J. (2015). Total Synthesis of Amphidinolide K, a Macrolide That Stabilizes F-Actin. J. Org. Chem..

[8] Trigili C., Pera B., Barbazanges M., Cossy J., Meyer C., Pineda O., Rodríguez-Escrich C., Urpí F., Vilarrasa J., Díaz J.F. (2011). Mechanism of Action of the Cytotoxic Macrolides Amphidinolide X and J. ChemBioChem.

[9] Bauer I., Maranda L., Young K.A., Shimizu Y., Fairchild C., Cornell L., MacBeth J., Huang S. (1995). Isolation and Structure of Caribenolide I, a Highly Potent Antitumor Macrolide from a Cultured Free-Swimming Caribbean Dinoflagellate, *Amphidinium* sp. S1-36-5. J. Org. Chem..

[10] Tsuda M., Oguchi K., Iwamoto R., Okamoto Y., Kobayashi J., Fukushi E., Kawabata J., Ozawa T., Masuda A., Kitaya Y. (2007). Iriomoteolide-1a, a Potent Cytotoxic 20-Membered Macrolide from a Benthic Dinoflagellate *Amphidinium* Species. J. Org. Chem..

[11] Kumagai K., Tsuda M., Fukushi E., Kawabata J., Masuda A., Tsuda M. (2017). Iriomoteolides-9a and 11a: Two new odd-numbered macrolides from the marine dinoflagellate *Amphidinium* species. J. Nat. Med..

[12] Takahashi Y., Kubota T., Kobayashi J. (2007). Amphidinolactone B, a new 26-membered macrolide from dinoflagellate *Amphidinium* sp.. J. Antibiot..

[13] Oguchi K., Tsuda M., Iwamoto R., Okamoto Y., Kobayashi J., Fukushi E., Kawabata J., Ozawa T., Masuda A., Kitaya Y. (2008). Iriomoteolide-3a, a Cytotoxic 15-Membered Macrolide from a Marine Dinoflagellate *Amphidinium* Species. J. Org. Chem..

[14] Kubota T., Endo T., Takahashi Y., Tsuda M., Kobayashi J. (2006). Amphidinin B, a new polyketide metabolite from marine dinoflagellate *Amphidinium* sp.. J. Antibiot..

[15] Kubota T., Sato H., Iwai T., Kobayashi J. (2016). Biosynthetic Study of Amphidinin A and Amphidinolide P. Chem. Pharm. Bull..

[16] Iwai T., Kubota T., Kobayashi J. (2014). Absolute Configuration of Amphidinin A. J. Nat. Prod..

[17] Kubota T., Iwai T., Sakai K., Gonoi T., Kobayashi J. (2014). Amphidinins C–F, Amphidinolide Q Analogues from Marine Dinoflagellate *Amphidinium* sp.. Org. Lett..

[18] Sousa T.S., Nuzzo G., Torres M.C.M., Lopes N.P., Cutignano A., Jimenez P.C., Santos E.A., Gomes B.A., Sardo A., Pessoa O.D.L. (2015). Amphidinolide P from the Brazilian octocoral *Stragulum bicolor*. Rev. Bras. Farmacogn..

[19] Nuzzo G., Gomes B.A., Luongo E., Torres M.C.M., Santos E.A., Cutignano A., Pessoa O.D.L., Costa-Lotufo L.V., Fontana A. (2016). Dinoflagellate-Related Amphidinolides from the Brazilian Octocoral *Stragulum bicolor*. J. Nat. Prod..

[20] Ishibashi M., Takahashi M., Kobayashi J. (1995). Amphidinolides O and P, Novel 15-Membered Macrolides from the Dinoflagellate *Amphidinium* sp.: Analysis of the Relative Stereochemistry and Stable Solution Conformation. J. Org. Chem..

[21] Cutignano A., Nuzzo G., Ianora A., Luongo E., Romano G., Gallo C., Sansone C., Aprea S., Mancini F., D’Oro U. (2015). Development and application of a novel SPE-method for bioassay-guided fractionation of marine extracts. Mar. Drugs.

[22] Trost B.M., Papillon J.P.N., Nussbaumer T. (2005). Ru-catalyzed alkene-alkyne coupling. Total synthesis of amphidinolide P. J. Am. Chem. Soc..

[23] Williams D.R., Myers B.J., Mi L., Binder R.J. (2013). Studies for the total synthesis of amphidinolide P. J. Org. Chem..

[24] Hwang M.H., Han S.J., Lee D.H. (2013). Convergent and enantioselective total synthesis of (-)-amphidinolide O and (-)-amphidinolide P. Org. Lett..

[25] Li Y., Carbone M., Vitale R.M., Amodeo P., Castelluccio F., Sicilia G., Mollo E., Nappo M., Cimino G., Guo Y.-W. (2010). Rare Casbane Diterpenoids from the Hainan Soft Coral *Sinularia depressa*. J. Nat. Prod..

[26] Gavagnin M., Carbone M., Amodeo P., Mollo E., Vitale R.M., Roussis V., Cimino G. (2007). Structure and Absolute Stereochemistry of Syphonoside, a Unique Macrocyclic Glycoterpenoid from Marine Organisms. J. Org. Chem..

[27] Fontana A., Ciavatta M.L., Amodeo P., Cimino G. (1999). Single solution phase conformation of new antiproliferative cembranes. Tetrahedron.

[28] Kobayashi J., Takahashi M., Ishibashi M. (1996). Amphidinolide Q, a novel 12-membered macrolide from the cultured marine dinoflagellate *Amphidinium* sp.. Tetrahedron Lett..

