# Supplementary material for: Potent Cytotoxic Analogs of Amphidinolides from the Atlantic Octocoral Stragulum bicolor
Source: Mar Drugs. 2019 Jan 16;17(1):58. doi: 10.3390/md17010058 (PMC6356882; doi:10.3390/md17010058)
Supplement: Supplementary file 1 [file marinedrugs-17-00058-s001.pdf]

SUPPLYMENTARY MATERIAL

Potent Cytotoxic Analogs of Amphidinolides from the Atlantic Octocoral *Stragulum bicolor*

Genoveffa Nuzzo [1,†,*], Bruno de Araújo Gomes [2,†], Carmela Gallo [1,†], Pietro Amodeo [1], Clementina Sansone [3], Otília D. L. Pessoa [2], Emiliano Manzo [1], Rosa Maria Vitale [1], Adrianna Ianora [3], Evelyne A. Santos [4], Leticia V. Costa-Lotufo [4] and Angelo Fontana [1,*]

[1] Institute of Biomolecular Chemistry, National Research Council (CNR), Pozzuoli 80078, Naples, Italy; carmen.gallo@icb.cnr.it (C.G.); pamodeo@icb.cnr.it (P.A.); emanzo@icb.cnr.it (E.M.); rmvitale@icb.cnr.it (R.M.V.)

[2] Departamento de Química Orgânica e Inorgânica, Universidade Federal do Ceará, Fortaleza, CE, Brazil; brunoaraujogomes1986@yahoo.com.br (B.A.G.); otilialoiola@gmail.com (O.D.L.P.)

[3] Stazione Zoologica Anton Dohrn, Villa Comunale, 80121, Napoli, Italy; clementina.sansone@szn.it (C.S.); ianora@szn.it (A.I.)

[4] Departamento de Farmacologia, Instituto de Ciências Biomédicas, Universidade de São Paulo, São Paulo, SP, Brazil; alvesevelyne@yahoo.com.br (E.A.S.); costalotufo@gmail.com (L.V.C.-L.)

† These authors have equally contributed to this work.

* Correspondence: nuzzo.genoveffa@icb.cnr.it (G.N.); angelo.fontana@icb.cnr.it (A.F.); Tel.: +39 081 8675104 (G.N.); +39 081 8675096 (A.F.)

Table S1. ^{1}H NMR data of compounds **1-4** in C_6D_6 (600 MHz**)**

	**1**	**2**	**3**	**4**
position	δ_H (*J* in Hz) [a]	δ_H (*J* in Hz) [a]	δ_H (*J* in Hz) [a]	δ_H (*J* in Hz)[a]
1				
2	3.97, m	5.04, dd (7.0, 5.0)	5.27, dd (8.0, 5.0)	4.14, ddd (10.0, 6.0, 3.0)
3	1.79, ddd (14.0, 8.0, 5.3)	2.03, dt (14.0, 7.0)	1.93, m	1.58, m
	1.53, ddd (14.0, 6.0, 3.0)	1.65, dt (14.0, 5.0)	1.73, m	1.45, ddd (14.0, 10.0, 4.0)
4	2.23, m	2.22, m	2.16, m	2.52, m
5	5.22, dd (15.5, 8.0)	5.27, dd (16.0, 7.0)	5.34, dd (16.0, 8.0)	5.21, dd (15.0, 8.0)
6	5.14, dt (15.5, 7.0)	5.19, dt (16.0, 7.0)	5.23, m	5.47, dt (15.0, 7.0)
7	2.49, m	2.47, ddd (14.0, 6.5, 3.0)	2.28, dt (15.0, 6.0)	2.24, m
	1.70, dt (14.0, 8.0)	1.76, m	1.98, m	2.04, m
8	2.63, m	2.66, ddd (7.5, 3.0, 2.0)	2.50, ddd (6.0, 5.0, 2.0)	2.61, td (5.0, 2.0)
9	2.53, m	2.54, m	2.72, ddd (6.0, 3.0, 2.0)	2.79, td (5.5, 2.0)
10	2.64, m	2.63, dd (12.0, 3.0)	2.62, dd (14.0, 3.0)	2.40, dd (16.0, 5.5)
	2.07, dd (13.5, 8.5)	2.10, dd (12.0, 8.5)	1.95, m	2.26, dd (16.0, 5.5)
12	6.28, d (16.0)	6.25, d (16.0)	5.73, d (11.5)	6.29, d (16.0)
13	5.47, dd (16.0, 8.0)	5.56, dd (16.0, 8.0)	5.38, dd (11.5, 10.0)	5.70, dd (16.0, 7.0)
14	5.37, dd (8.0, 7.5)	5.33, t (8.0)	6.03, dd (10.0, 7.0)	3.86, td (7.0, 2.5)
15	2.35, m	2.53, m	2.54, m	2.20, m
17	4.78, s	4.84, s	4.86, s	4.80, s
	4.77, s		4.85, s	
18	0.88, d (7.0)	0.82, d (7.0)	0.85, d (7.0)	0.89, d (7.0)
19	0.82, d (7.0)	0.95, d (7.0)	1.05, d (7.0)	0.90, d (7.0)
20	1.63, s	1.72, s	1.72, s	1.60, s
21	4.90, s	4.91, s	4.91, s	5.04, s
	4.80, s	4.83, s		
2-OH	2.70, d (5.7)			2.82, d (6.0)
14-OH				1.78, d (2.5)
OMe				3.26, s
OAc		1.73, s	1.74, s	

Table S2. ^{13}C NMR data[a] of compounds **1-4** in C_6D_6 (600 MHz**)**

	**1**	**2**	**3**	**4**
Position	δ_C	δ_C	δ_C	$\delta_{C\ type}$
1	174.6	169.7	n.d	176.1, C
2	70.3	72.4	71.8	68.9, CH
3	41.8	37.5	37.5	41.6, CH_2
4	34.3	34.4	34.5	33.5, CH
5	137.7	137.5	137.0	137.8, CH
6	122.7	122.3	124.2	125.0, CH
7	35.5	35.5	35.0	35.0, CH_2
8	59.3	58.7	57.3	57.7, CH
9	57.0	57.6	55.6	55.9, CH
10	36.6	36.3	39.5	35.0, CH_2
11	141.2	141.4	135.4	142.9, C
12	137.2	135.6	134.4	133.0, CH
13	126.5	126.8	127.4	131.9, CH
14	77.8	77.8	72.6	74.6, CH
15	45.0	44.7	46.1	47.9, CH
16	145.9	145.8	145.9	147.2, C
17	112.3	112.3	113.0	112.5, CH_2
18	21.7	21.6	22.3	21.2, CH_3
19	14.6	14.6	15.7	15.6, CH_3
20	19.4	19.7	20.1	19.0, CH_3
21	119.0	118.7	117.5	117.0, CH_2
$-OCH_3$				51.4, CH_3
Ac (C=O)		170.0	169.2	
Ac(CH_3)		19.3	20.1	

[a] ^{13}C data were obtained from HSQCed and HMBC.

Table S3. ^{1}H and ^{13}C NMR data of compounds **2** and **3** in $CDCl_3$ (600 MHz**).**

	**2**		**3**	
	$\delta_{H,}$ m (*J* in Hz)	δ_C	$\delta_{H,}$ m (*J* in Hz)	δ_C
1		169.6		168.5
2	4.87, dd (7.5, 4.4)	76.8	5.13, dd (7.0, 5.0)	71.5
3	1.96, dt (15.0, 7.5)	42.9	1.87, m	37.1
	1.66, dt (15.0, 4.5)			
4	2.37, m	38.6	2.34, m	34.9
5	5.50, dd (15.5, 7.5)	141.9	5.36, dd (16.0, 8.0)	137.3
6	5.36, dt (15.5, 7.0)	127.6	5.33, dt (16.0, 7.5)	123.8
7	2.62, overlapped	40.0	2.42, dt (15.0, 4.5)	34.6
	1.84, m		1.98, m	
8	2.81, dt (8.0, 2.2)	63.6	2.65, m	58.0
9	2.63, overlapped	61.8	2.75, m	56.5
10	2.77, dd (13.5, 3.0)	41.0	2.79, dd (14.5, 3.5)	39.5
	2.15, dd (13.5, 9.0)		2.10, dd (14.5, 6.5)	
11		136.0		139.7
12	6.36, d (16.0)	134.3	5.98, d (12.0)	134.3
13	5.57, dd (16.0, 8.0)	130.4	5.48, dd (12.0, 10.0)	127.5
14	5.20, t (8.0)	82.4	5.84, dd (10.0, 7.0)	72.3
15	2.55, m	49.4	2.55, m	45.7
16		145.5		145.5
17	4.81, s	116.3	4.82, s	113.0
	4.75, s		4.78, s	
18	1.03, d (7.0)	26.5	1.03, d (7.0)	22.5
19	1.01, d (7.0)	19.7	1.06, d (7.0)	15.8
20	1.72, s	24.4	1.73, s	20.0
21	5.18, s	124.0	5.15, s	118.0
	5.17, s		4.98, s	
Ac (C=O)		170.1		169.6
Ac(CH_3)	2.10, s	25.1	2.08, s	20.9

[a] ^{13}C data were obtained from HSQCed and HMBC.

Table S4. List of NMR-derived distance restraints corresponding to "surely-missing" NOE cross-peak (antiNOEs) for AMP-PX2 (**2**), used in SA/MD/EM calculations. Lower limit distances of 3.2 Å were imposed for all restraints, except for those involving methyl groups (**bold**), for which a 3.7 Å limit was instead used.

Atom pair	*Atom pair*	*Atom pair*	*Atom pair*	*Atom pair*
H2-H7a	H3a-H19b	H4-H19a	H7a-H19b	**H10a-Me20**
H2-H7b	**H3a-Me20**	H4-H19b	**H7a-Me18**	H10b-H14
H2-H8	H3b-H8	**H4-Me20**	**H7a-Me20**	H10b-H19b
H2-H9	H3b-H9	H5-H8	H7b-H12	**H10b-Me18**
H2-H10a	H3b-H10a	H5-H9	H7b-H13	**H10b-Me20**
H2-H10b	H3b-H10b	H5-H10a	H7b-H14	H12-H15
H2-H12	H3b-H12	H5-H10b	H7b-H17a	H12-H17a
H2-H13	H3b-H13	H5-H12	H7b-H17b	H12-H17b
H2-H14	H3b-H14	**H5-Me20**	H7b-H19a	H12-H19a
H2-H15	H3b-H15	H6-H9	H7b-H19b	**H12-Me18**
**H2-Me20**	H3b-H17a	H6-H10a	**H7b-Me18**	**H12-Me21**
H3a-H5	H3b-H17b	H6-H10b	**H7b-Me20**	**H13-Me18**
H3a-H6	H3b-H19a	H6-H12	H8-H12	**H13-Me21**
H3a-H8	H3b-H19b	H6-H15	H8-H14	H15-H19b
H3a-H9	**H3b-Me20**	**H6-Me20**	H8-H19b	**H15-Me18**
H3a-H12	H4-H8	H7a-H12	**H8-Me20**	**H17a-Me18**
H3a-H13	H4-H12	H7a-H13	H9-H12	**H17b-Me18**
H3a-H14	H4-H13	H7a-H14	H9-H19b	**Me18-H19a**
H3a-H17a	H4-H14	H7a-H17a	H10a-H14	**Me18-H19b**
H3a-H17b	H4-H17a	H7a-H17b	H10a-H19b	**H19b-Me20**
H3a-H19a	H4-H17b	H7a-H19a	**H10a-Me18**	**H19b-Me21**

Table S5. NMR-derived distance range restraints only involving the macrocyclic structure and the groups rigidly anchored to it for AMP-PX2 (**2**) (*italics*) and the corresponding distances (with restraint violations in parenthesis) in the final rEM representative conformers for the 4S diastereomer. Average distances, violations and conformer weights corresponding to the lowest total average violation ensemble are also shown in bold. All distances and violations in Å.

			Conformer					
At. pair	***Restraint***	***Ensemble***	***I***	***II***	***III***	***IV***	***V***	***VI***
i – j	***range (Å)***	***r_{avg}(viol) (Å)***	***r (viol) (Å)***	***r (viol) (Å)***	***r (viol) (Å)***	***r (viol) (Å)***	***r (viol) (Å)***	***r (viol) (Å)***
H2 - H4	*2.51 - 3.21*	**2.63 (0)**	2.69 (0)	4.28 (1.07)	2.34 (-0.17)	2.36 (-0.15)	2.85 (0)	2.47 (-0.04)
H2 - Me18	*3.18 - 3.88*	**3.88 (0)**	4.64 (0.76)	4.39 (0.51)	3.17 (-0.01)	3.09 (-0.09)	4.72 (0.84)	4.58 (0.70)
H4 - H5	*2.03 - 2.73*	**2.45 (0)**	2.33 (0)	3.08 (0.35)	2.99 (0.26)	2.81 (0.08)	2.33 (0)	2.32 (0)
H4 - H6	*2.90 - 3.60*	**2.95 (0)**	3.82 (0.22)	2.38 (-0.52)	2.63 (-0.27)	2.96 (0)	3.82 (0.22)	3.83 (0.23)
H4 - Me18	*2.42 - 3.12*	**2.59 (0)**	2.60 (0)	2.57 (0)	2.60 (0)	2.58 (0)	2.60 (0)	2.60 (0)
H5 - Me18	*2.84 - 3.54*	**3.30 (0)**	3.63 (0.09)	2.94 (0)	3.56 (0.02)	2.73 (-0.11)	3.55 (0.01)	3.48 (0)
H6 - H8	*3.11 - 3.81*	**3.15 (0)**	3.24 (0)	2.85 (-0.26)	3.00 (-0.11)	3.42 (0)	3.67 (0)	3.33 (0)
H6 - Me18	*2.68 - 3.38*	**3.02 (0)**	2.87 (0)	4.18 (0.80)	3.02 (0)	4.73 (1.35)	2.94 (0)	3.09 (0)
H8 - H13	*1.82 - 2.52*	**2.47 (0)**	2.37 (0)	2.24 (0)	3.95 (1.43)	3.12 (0.60)	5.98 (3.46)	3.47 (0.95)
H12 - H13	*2.64 - 3.34*	**3.09 (0)**	3.09 (0)	3.09 (0)	3.09 (0)	3.09 (0)	3.08 (0)	3.09 (0)
H12 - H14	*2.32 - 3.02*	**2.45 (0)**	2.45 (0)	2.37 (0)	2.43 (0)	2.49 (0)	3.71 (0.69)	2.43 (0)
H13 - H14	*2.45 - 3.15*	**2.97 (0)**	3.05 (0)	3.09 (0)	3.06 (0)	3.01 (0)	2.37 (-0.08)	3.09 (0)
H2 - H3b	*2.65 - 3.35*	**2.68 (0)**	2.72 (0)	2.36 (-0.29)	3.03 (0)	3.03 (0)	2.61 (-0.05)	2.98 (0)
H2 - H3a	*2.36 - 3.06*	**2.82 (0)**	3.04 (0)	2.49 (0)	2.71 (0)	2.71 (0)	3.06 (0)	2.85 (0)
H3b - H5	*2.99 - 3.69*	**3.09 (0)**	3.65 (0)	3.96 (0.27)	2.30 (-0.70)	3.58 (0)	3.69 (0)	3.87 (0.18)
H3b - H6	*1.87 - 2.57*	**2.58 (0)**	2.50 (0)	4.50 (1.93)	4.52 (1.95)	2.24 (0)	2.43 (0)	2.48 (0)
H3a - Me18	*2.48 - 3.18*	**2.87 (0)**	2.84 (0)	3.97 (0.79)	2.72 (0)	2.72 (0)	2.79 (0)	2.85 (0)
H3a - H4	*1.95 - 2.65*	**2.57 (0)**	2.56 (0)	2.38 (0)	3.01 (0.36)	3.02 (0.37)	2.59 (0)	2.46 (0)
H3b - Me18	*3.00 - 3.70*	**3.08 (0)**	3.03 (0)	2.89 (-0.11)	3.82 (0.12)	3.83 (0.13)	3.08 (0)	2.94 (-0.06)
H5 - H7a	*2.55 - 3.25*	**2.92 (0)**	3.50 (0.25)	2.47 (-0.09)	2.43 (-0.12)	3.65 (0.40)	3.65 (0.40)	3.63 (0.38)
H5 - H7b	*1.83 - 2.53*	**2.55 (0.01)**	2.42 (0)	3.64 (1.11)	3.55 (1.02)	2.47 (0)	2.48 (0)	2.45 (0)
H6 - H7a	*1.99 - 2.69*	**2.62 (0)**	2.56 (0)	3.08 (0.39)	3.11 (0.42)	2.48 (0)	2.44 (0)	2.46 (0)
H7b - H8	*2.09 - 2.79*	**2.59 (0)**	2.58 (0)	2.92 (0.13)	2.44 (0)	2.53 (0)	2.47 (0)	2.67 (0)
H10a - H19a	*2.20 - 2.90*	**2.44 (0)**	2.42 (0)	2.45 (0)	2.41 (0)	2.37 (0)	2.41 (0)	3.67 (0.77)
H10b - H12	*2.83 - 3.53*	**3.48 (0)**	3.85 (0.32)	3.77 (0.24)	3.80 (0.27)	3.85 (0.32)	2.42 (-0.42)	3.60 (0.07)
H10b - H13	*2.08 - 2.78*	**2.25 (0)**	2.16 (0)	2.26 (0)	2.30 (0)	2.38 (0)	4.67 (1.89)	4.95 (2.17)
H10b - H19a	*3.07 - 3.77*	**3.38 (0)**	3.66 (0)	3.68 (0)	3.60 (0)	3.55 (0)	3.63 (0)	2.47 (-0.60)
H12 - H19b	*2.18 - 2.88*	**2.44 (0)**	2.41 (0)	2.38 (0)	2.40 (0)	2.38 (0)	3.83 (0.95)	3.82 (0.94)
H2 - H7a	*3.50 - ∞*	**5.65 (0)**	5.37 (0)	6.36 (0)	6.67 (0)	6.71 (0)	5.49 (0)	5.60 (0)
H2 - H9	*3.50 - ∞*	**6.54 (0)**	6.48 (0)	7.78 (0)	6.17 (0)	7.21 (0)	5.76 (0)	6.68 (0)
H3a - H12	*3.50 - ∞*	**5.72 (0)**	5.39 (0)	6.28 (0)	7.34 (0)	7.69 (0)	5.48 (0)	6.45 (0)
H3a - H13	*3.50 - ∞*	**5.27 (0)**	6.43 (0)	4.10 (0)	5.60 (0)	5.58 (0)	6.23 (0)	6.99 (0)
H3a - H14	*3.50 - ∞*	**5.11 (0)**	5.04 (0)	4.85 (0)	5.75 (0)	5.74 (0)	4.79 (0)	5.81 (0)
H3a - H19a	*3.50 - ∞*	**8.55 (0)**	8.24 (0)	8.58 (0)	9.52 (0)	10.08 (0)	8.59 (0)	9.40 (0)
H3a - H19b	*3.50 - ∞*	**7.61 (0)**	7.18 (0)	8.15 (0)	9.13 (0)	9.71 (0)	7.70 (0)	8.69 (0)
H3b - H8	*3.50 - ∞*	**4.63 (0)**	4.32 (0)	5.78 (0)	6.42 (0)	5.40 (0)	4.90 (0)	4.75 (0)
H3b - H12	*3.50 - ∞*	**4.18 (0)**	3.85 (0)	7.52 (0)	5.73 (0)	6.16 (0)	3.99 (0)	5.07 (0)
H3b - H13	*3.50 - ∞*	**4.84 (0)**	4.89 (0)	5.72 (0)	4.38 (0)	4.31 (0)	4.90 (0)	6.22 (0)

H3b - H14	*3.50 - ∞*	**4.16 (0)**	3.94 (0)	5.90 (0)	4.38 (0)	4.36 (0)	4.04 (0)	4.91 (0)
H3b - H19a	*3.50 - ∞*	**7.02 (0)**	6.56 (0)	9.94 (0)	7.91 (0)	8.51 (0)	6.88 (0)	8.21 (0)
H3b - H19b	*3.50 - ∞*	**6.09 (0)**	5.62 (0)	9.39 (0)	7.44 (0)	8.09 (0)	6.12 (0)	7.70 (0)
H4 - H12	*3.50 - ∞*	**6.54 (0)**	6.76 (0)	7.67 (0)	5.84 (0)	6.04 (0)	5.90 (0)	6.88 (0)
H4 - H13	*3.50 - ∞*	**4.22 (0)**	6.89 (0)	5.48 (0)	3.22 (-0.28)	3.32 (-0.18)	7.70 (0)	6.84 (0)
H4 - H14	*3.50 - ∞*	**5.64 (0)**	6.54 (0)	6.72 (0)	4.58 (0)	4.58 (0)	6.62 (0)	6.75 (0)
H4 - H19a	*3.50 - ∞*	**8.73 (0)**	9.00 (0)	9.21 (0)	7.83 (0)	8.20 (0)	9.30 (0)	8.71 (0)
H4 - H19b	*3.50 - ∞*	**8.33 (0)**	8.42 (0)	9.09 (0)	7.65 (0)	7.97 (0)	8.86 (0)	8.17 (0)
H5 - H8	*3.50 - ∞*	**3.74 (0)**	3.81 (0)	3.75 (0)	4.65 (0)	3.36 (-0.14)	3.48 (-0.02)	3.40 (-0.10)
H5 - H9	*3.50 - ∞*	**4.30 (0)**	5.49 (0)	4.58 (0)	3.19 (-0.31)	5.29 (0)	5.29 (0)	5.51 (0)
H5 - H10a	*3.50 - ∞*	**6.76 (0)**	7.28 (0)	6.42 (0)	6.01 (0)	6.52 (0)	6.88 (0)	6.67 (0)
H5 - H10b	*3.50 - ∞*	**5.94 (0)**	6.47 (0)	5.59 (0)	5.33 (0)	5.37 (0)	5.78 (0)	7.01 (0)
H5 - H12	*3.50 - ∞*	**5.98 (0)**	6.38 (0)	5.36 (0)	6.01 (0)	7.32 (0)	5.02 (0)	5.97 (0)
H6 - H9	*3.50 - ∞*	**3.49 (-0.01)**	3.39 (-0.11)	4.93 (0)	3.82 (0)	3.17 (-0.33)	3.19 (-0.31)	3.88 (0)
H6 - H10a	*3.50 - ∞*	**5.36 (0)**	5.30 (0)	6.37 (0)	5.24 (0)	5.26 (0)	5.51 (0)	4.94 (0)
H6 - H10b	*3.50 - ∞*	**4.81 (0)**	5.01 (0)	5.37 (0)	4.16 (0)	4.28 (0)	5.03 (0)	5.87 (0)
H7a - H12	*3.50 - ∞*	**5.63 (0)**	5.72 (0)	5.40 (0)	6.48 (0)	5.90 (0)	5.03 (0)	5.14 (0)
H7a - H14	*3.50 - ∞*	**6.60 (0)**	6.93 (0)	6.03 (0)	6.81 (0)	5.82 (0)	7.62 (0)	6.88 (0)
H7a - H19a	*3.50 - ∞*	**5.64 (0)**	5.57 (0)	5.31 (0)	6.20 (0)	6.21 (0)	5.99 (0)	5.82 (0)
H7a - H19b	*3.50 - ∞*	**6.17 (0)**	6.14 (0)	5.66 (0)	6.90 (0)	6.66 (0)	6.61 (0)	6.47 (0)
H7b - H12	*3.50 - ∞*	**6.15 (0)**	6.11 (0)	6.70 (0)	7.43 (0)	6.94 (0)	5.07 (0)	5.56 (0)
H7b - H14	*3.50 - ∞*	**6.92 (0)**	6.70 (0)	7.27 (0)	7.73 (0)	6.73 (0)	7.90 (0)	7.01 (0)
H7b - H19a	*3.50 - ∞*	**6.55 (0)**	6.70 (0)	6.21 (0)	6.93 (0)	7.12 (0)	7.01 (0)	5.59 (0)
H7b - H19b	*3.50 - ∞*	**7.07 (0)**	7.09 (0)	6.92 (0)	7.85 (0)	7.76 (0)	7.54 (0)	6.04 (0)
H8 - H12	*3.50 - ∞*	**4.03 (0)**	4.13 (0)	4.83 (0)	6.06 (0)	5.59 (0)	3.13 (-0.37)	3.20 (-0.30)
H8 - H14	*3.50 - ∞*	**4.86 (0)**	4.59 (0)	5.14 (0)	6.87 (0)	5.96 (0)	6.58 (0)	4.67 (0)
H9 - H12	*3.50 - ∞*	**4.19 (0)**	4.49 (0)	4.54 (0)	3.78 (0)	3.82 (0)	3.65 (0)	4.00 (0)
H10a - H19b	*3.50 - ∞*	**3.73 (0)**	3.71 (0)	3.71 (0)	3.71 (0)	3.69 (0)	3.71 (0)	4.25 (0)
H10b - H19b	*3.50 - ∞*	**4.20 (0)**	4.26 (0)	4.26 (0)	4.21 (0)	4.19 (0)	4.23 (0)	3.73 (0)
H10a - H14	*3.50 - ∞*	**5.85 (0)**	6.00 (0)	5.99 (0)	6.03 (0)	6.03 (0)	6.76 (0)	4.73 (0)
H10b - H14	*3.50 - ∞*	**4.97 (0)**	4.88 (0)	4.88 (0)	4.94 (0)	5.01 (0)	5.98 (0)	5.93 (0)
H12 - H19a	*3.50 - ∞*	**3.75 (0)**	3.71 (0)	3.70 (0)	3.71 (0)	3.70 (0)	4.29 (0)	4.29 (0)
H13 - Me18	*4.00 - ∞*	**6.68 (0)**	7.42 (0)	6.82 (0)	5.62 (0)	5.70 (0)	7.81 (0)	8.43 (0)
H3a - H5	*3.50 - ∞*	**3.74 (0)**	4.20 (0)	3.41 (-0.09)	3.13 (-0.37)	3.76 (0)	4.26 (0)	4.23 (0)
H3a - H6	*3.50 - ∞*	**3.89 (0)**	3.93 (0)	3.54 (0)	4.86 (0)	3.75 (0)	3.86 (0)	3.96 (0)
H3a - H7a	*3.50 - ∞*	**5.81 (0)**	6.26 (0)	5.16 (0)	5.50 (0)	5.51 (0)	6.17 (0)	6.26 (0)
H3a - H9	*3.50 - ∞*	**6.79 (0)**	7.17 (0)	6.72 (0)	5.84 (0)	6.90 (0)	6.67 (0)	7.65 (0)
H3b - H9	*3.50 - ∞*	**5.30 (0)**	5.50 (0)	8.02 (0)	4.38 (0)	5.32 (0)	4.95 (0)	6.07 (0)
H3b - H10a	*3.50 - ∞*	**7.00 (0)**	6.79 (0)	9.15 (0)	7.22 (0)	7.46 (0)	6.72 (0)	6.39 (0)
H3b - H10b	*3.50 - ∞*	**6.30 (0)**	6.10 (0)	7.74 (0)	6.26 (0)	6.33 (0)	5.88 (0)	7.60 (0)
H4 - H8	*3.50 - ∞*	**5.09 (0)**	5.52 (0)	4.60 (0)	5.14 (0)	4.40 (0)	5.45 (0)	5.14 (0)
H6 - H12	*3.50 - ∞*	**4.38 (0)**	4.10 (0)	6.99 (0)	6.51 (0)	4.72 (0)	4.12 (0)	4.50 (0)
H7a - H10b	*3.50 - ∞*	**4.69 (0)**	4.74 (0)	4.54 (0)	4.72 (0)	4.60 (0)	4.72 (0)	4.80 (0)
H7a - H13	*3.50 - ∞*	**4.84 (0)**	5.26 (0)	4.24 (0)	4.70 (0)	4.22 (0)	6.95 (0)	6.50 (0)
H7b - H13	*3.50 - ∞*	**4.99 (0)**	4.94 (0)	4.91 (0)	5.13 (0)	4.58 (0)	7.56 (0)	6.04 (0)
H7a - Me18	*4.00 - ∞*	**4.91 (0)**	4.80 (0)	4.98 (0)	5.07 (0)	6.12 (0)	4.76 (0)	4.85 (0)
H7b - Me18	*4.00 - ∞*	**5.08 (0)**	5.08 (0)	5.53 (0)	4.89 (0)	5.17 (0)	4.82 (0)	4.83 (0)
H8 - H19b	*3.50 - ∞*	**5.12 (0)**	5.40 (0)	5.73 (0)	6.23 (0)	6.22 (0)	5.99 (0)	3.66 (0)
H9 - H19b	*3.50 - ∞*	**4.19 (0)**	4.19 (0)	4.00 (0)	4.26 (0)	4.12 (0)	4.10 (0)	5.37 (0)

H10a - H12	*3.50* - ∞	**3.50 (0)**	4.31 (0)	4.26 (0)	4.32 (0)	4.33 (0)	3.64 (0)	2.32 (-1.18)
H10a - Me18	*4.00* - ∞	**8.45 (0)**	8.31 (0)	9.36 (0)	8.25 (0)	8.66 (0)	8.63 (0)	8.20 (0)
H10b - Me18	*4.00* - ∞	**7.84 (0)**	8.04 (0)	8.42 (0)	7.01 (0)	7.17 (0)	7.99 (0)	9.09 (0)
H12 - Me18	*4.00* - ∞	**6.71 (0)**	6.28 (0)	8.08 (0)	8.27 (0)	8.52 (0)	6.68 (0)	7.47 (0)
Me18 - H19a	*4.00* - ∞	**8.44 (0)**	7.90 (0)	9.75 (0)	9.87 (0)	10.39 (0)	9.00 (0)	9.70 (0)
Me18 - H19b	*4.00* - ∞	**7.87 (0)**	7.30 (0)	9.35 (0)	9.91 (0)	10.36 (0)	8.62 (0)	9.54 (0)
		**Weights**	**0.55**	**0.14**	**0.12**	**0.08**	**0.05**	**0.06**

Table S6. Dihedral angles of the macrocycle in the selected conformers of the 4S diastereomer of AMP-PX2 (**2**).

Dihedral[a]	**I**	**II**	**III**	**IV**	**V**	**VI**
O14-C1-C2-C3	73.9	74.1	63.2	53.4	75.6	-175.6
C1-C2-C3-C4	-146.9	74.8	-87.8	-86.8	-164.1	-108.4
C2-C3-C4-C5	67.4	-58.8	138.8	140.6	72.5	59.3
C3-C4-C5-C6	65.3	-109.9	-165.5	-48.6	61.8	56.3
C4-C5-C6-C7	179.3	-178.3	177.9	-173.9	172.1	173.6
C5-C6-C7-C8	118.4	-101.7	-113.0	99.2	102.0	100.7
C6-C7-C8-C9	98.8	152.1	69.2	87.0	83.5	116.3
C7-C8-C9-C10	-156.2	-153.1	-150.9	-148.9	-157.7	-158.3
C8-C9-C10-C11	86.2	103.5	171.8	141.1	113.9	-9.8
C9-C10-C11-C12	-82.8	-91.3	-75.7	-70.0	-80.2	85.5
C10-C11-C12-C13	-15.7	7.1	0.2	-4.8	178.8	-164.2
C11-C12-C13-C14	175.2	-178.8	179.2	-179.8	-177.5	177.2
C12-C13-C14-O14	-86.9	-131.9	-141.4	-155.6	37.0	-98.5
C13-C14-O14-C1	165.1	72.9	141.1	151.7	167.6	77.5
C14-O14-C1-C2	179.0	164.7	177.3	173.2	178.9	176.3

[a] The four atoms defining the dihedral angle are reported.

Table S7. Cytotoxic activities of compounds **2** and **3** by IC_{50} values in human adenocarcinoma colon (HCT-116), breast (MCF-7) and melanoma (SK-MEL-19) cancer cell lines.

	IC_{50} (ug.mL^{-1})		
	**HCT-116**	**MCF-7**	**SK-MEL-19**
**AMP-PX2 (2)**	> 13	> 13	> 13
**AMP-PX3 (3)**	> 13	> 13	> 13
DOX	0.02<br>0.016 - 0.027	0.02<br>0.01 – 0.03	0.1<br>0.08 – 0.21

[a]Data are presented as IC_{50} values (ug.mL^{-1}) and 95% confidence interval (CI95%) obtained by nonlinear regression for all cell lines from three independent experiments. [b]DOX denotes denotes the positive control, doxorubicin.

Figure S1 ^{1}H NMR spectrum of **1** (600 MHz, C_6D_6)

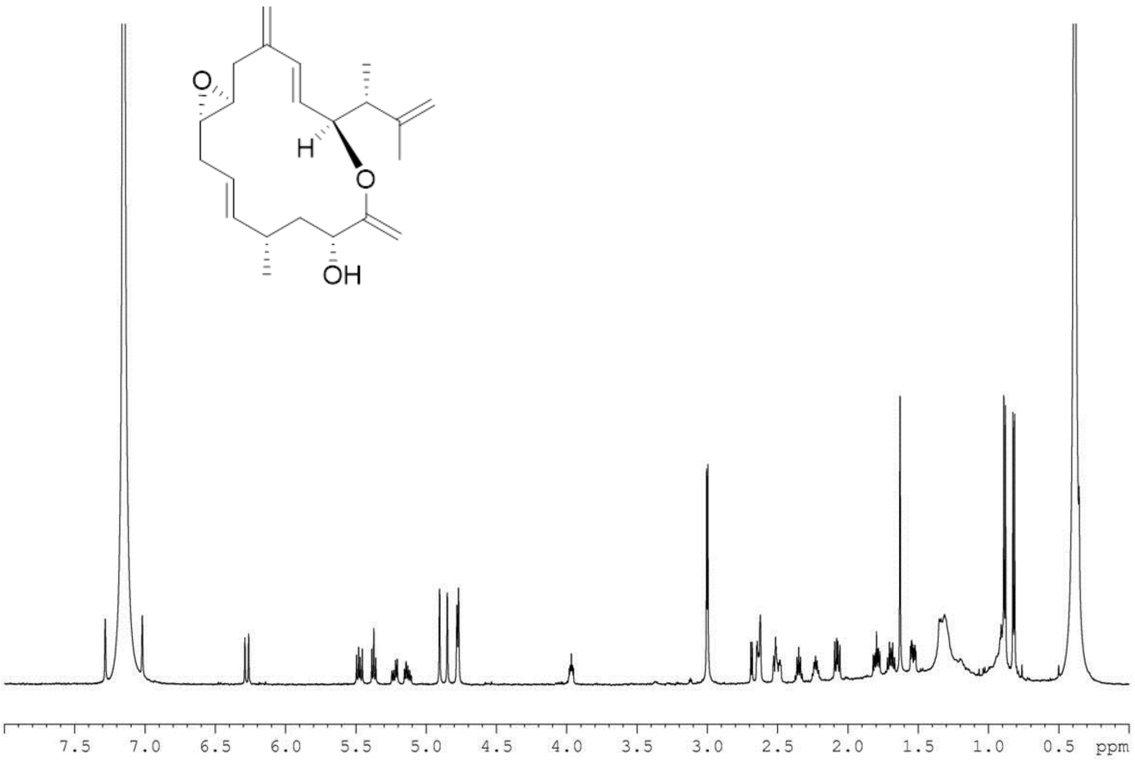

Figure S2 COSY NMR spectrum of **1** (600 MHz, C_6D_6)

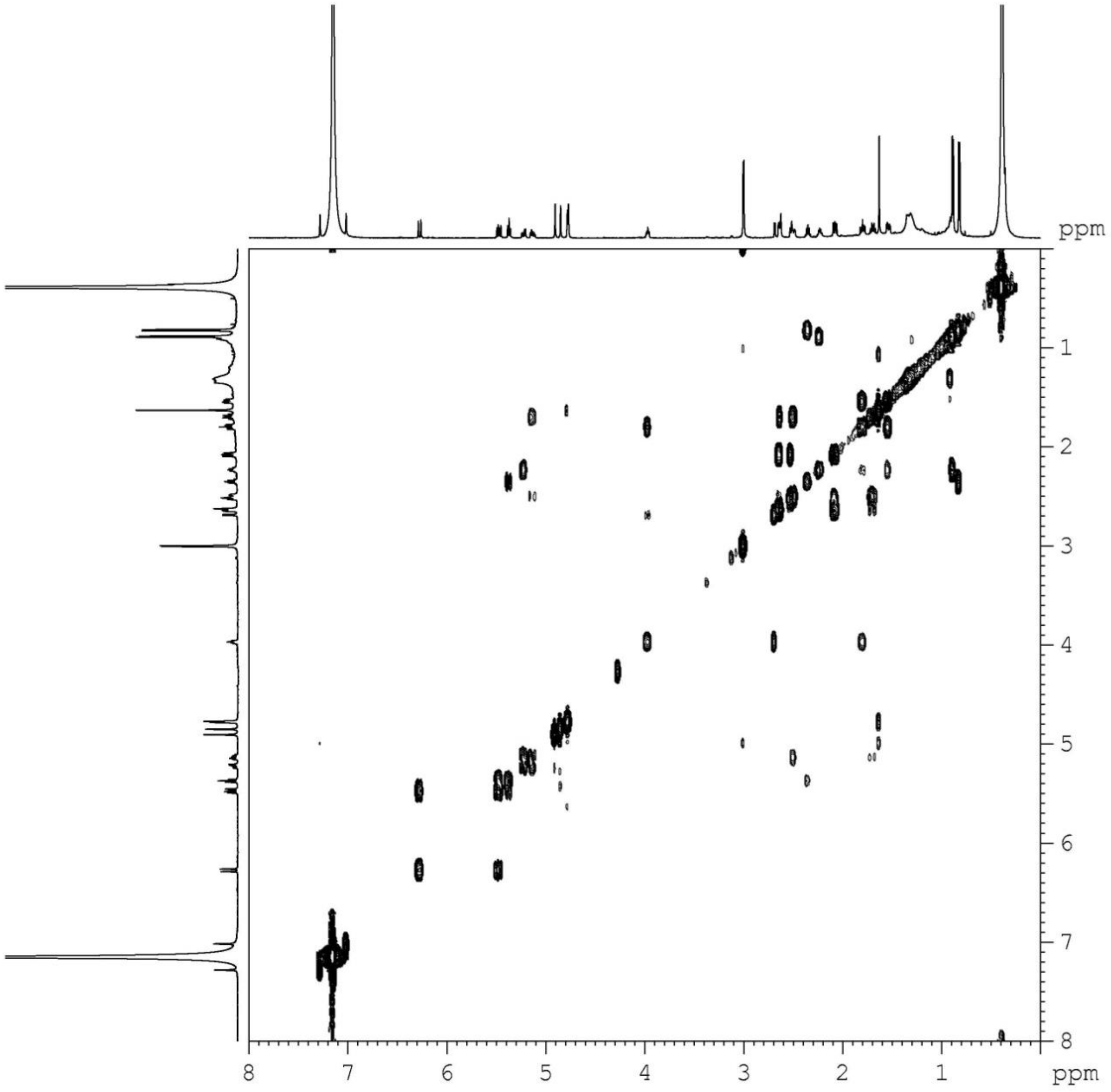

Figure S3 TOCSY NMR spectrum of **1** (600 MHz, C_6D_6)

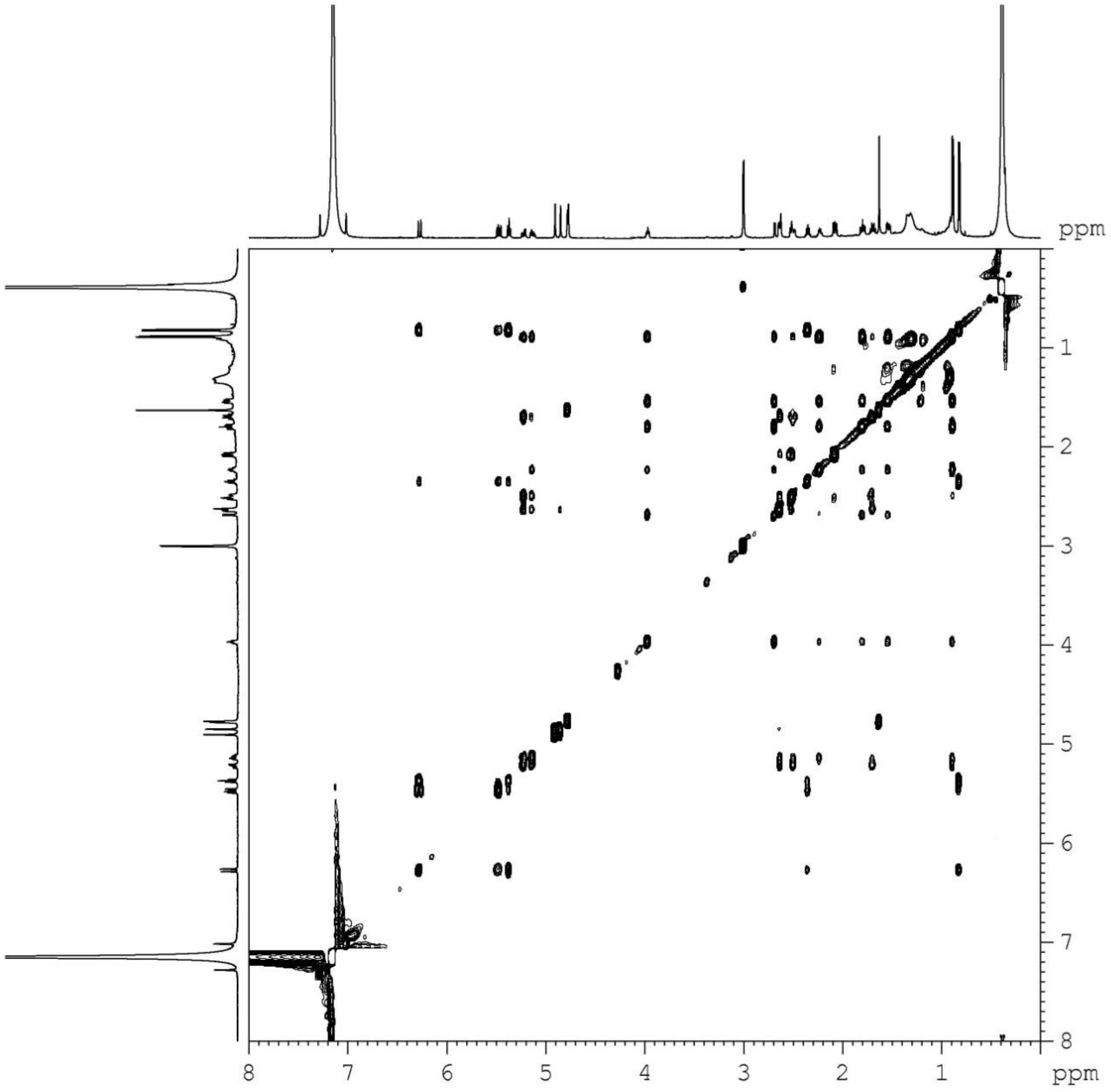

Figure S4 HSQCed NMR spectrum of **1** (600 MHz, C_6D_6)

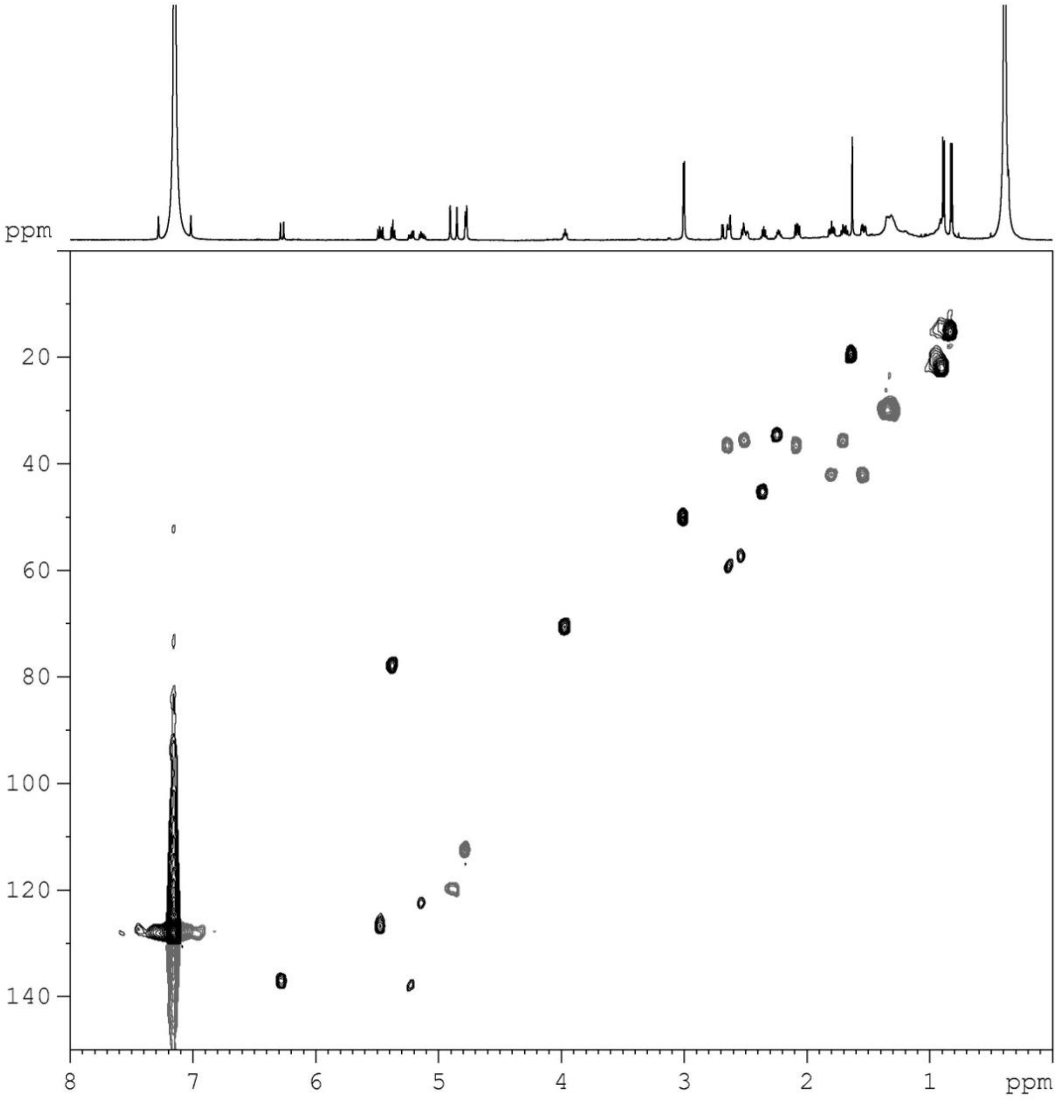

Figure S5 HMBC NMR spectrum of **1** (600 MHz, C_6D_6)

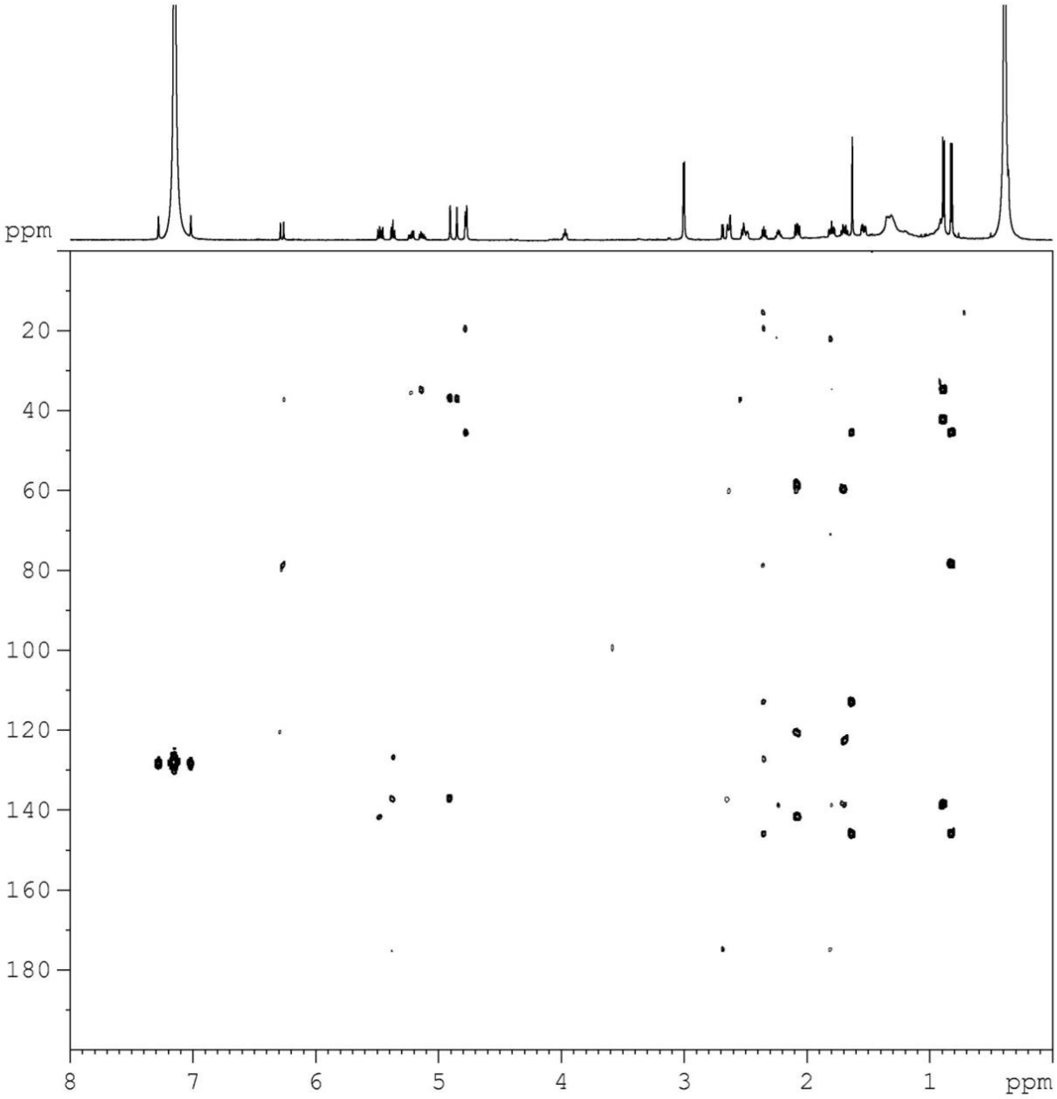

Figure S6 ^{1}H NMR spectrum of **2** (600 MHz, C_6D_6)

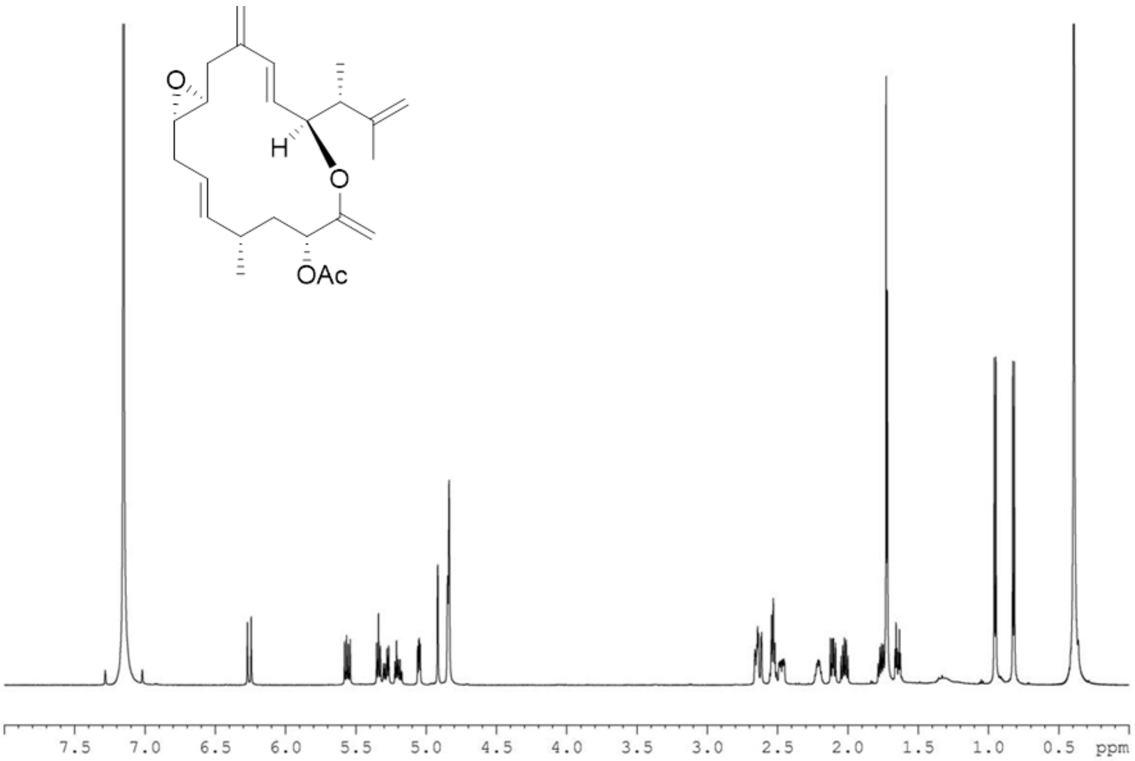

Figure S7 COSY NMR spectrum of **2** (600 MHz, C_6D_6)

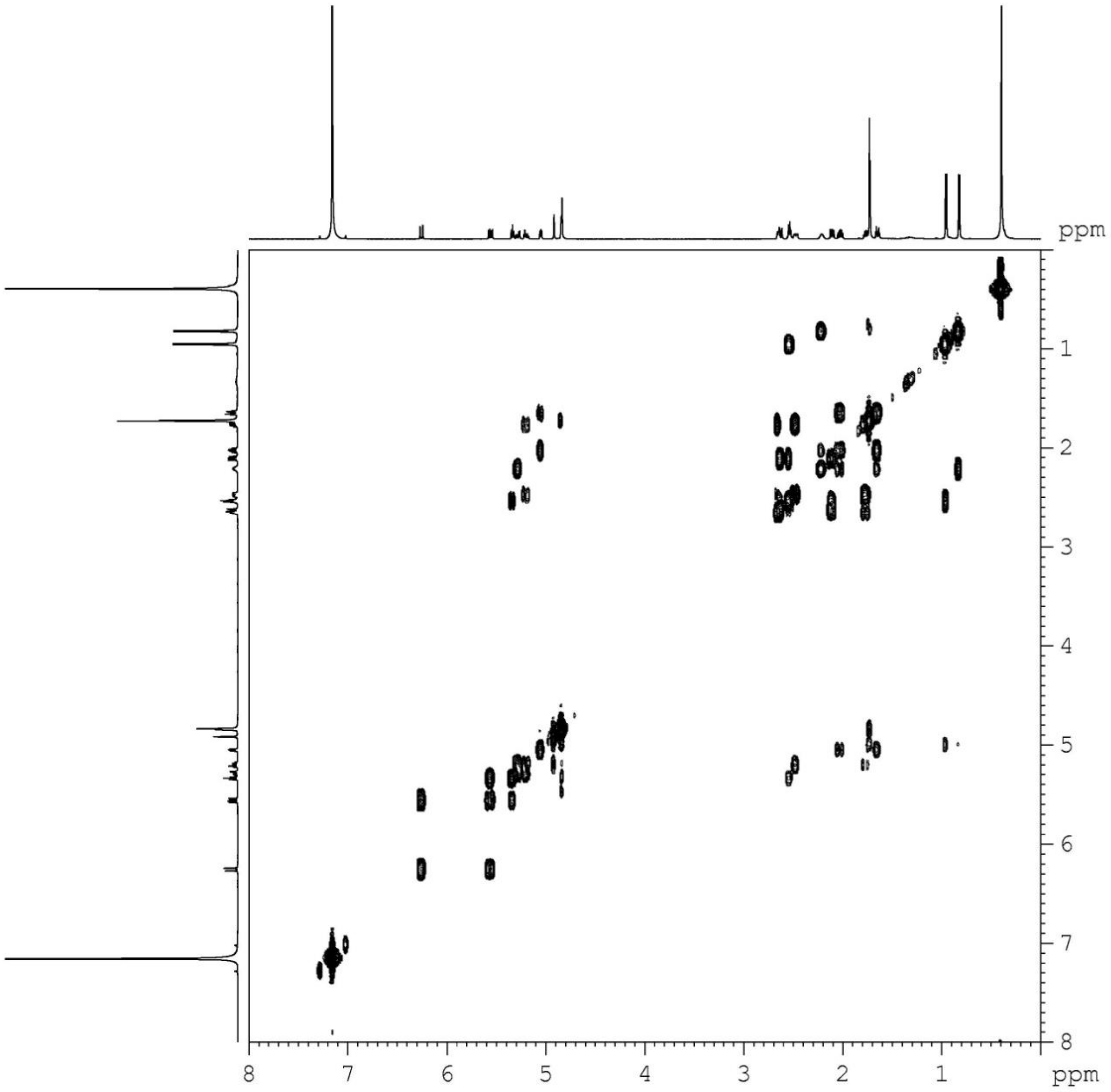

Figure S8 TOCSY NMR spectrum of **2** (600 MHz, C_6D_6)

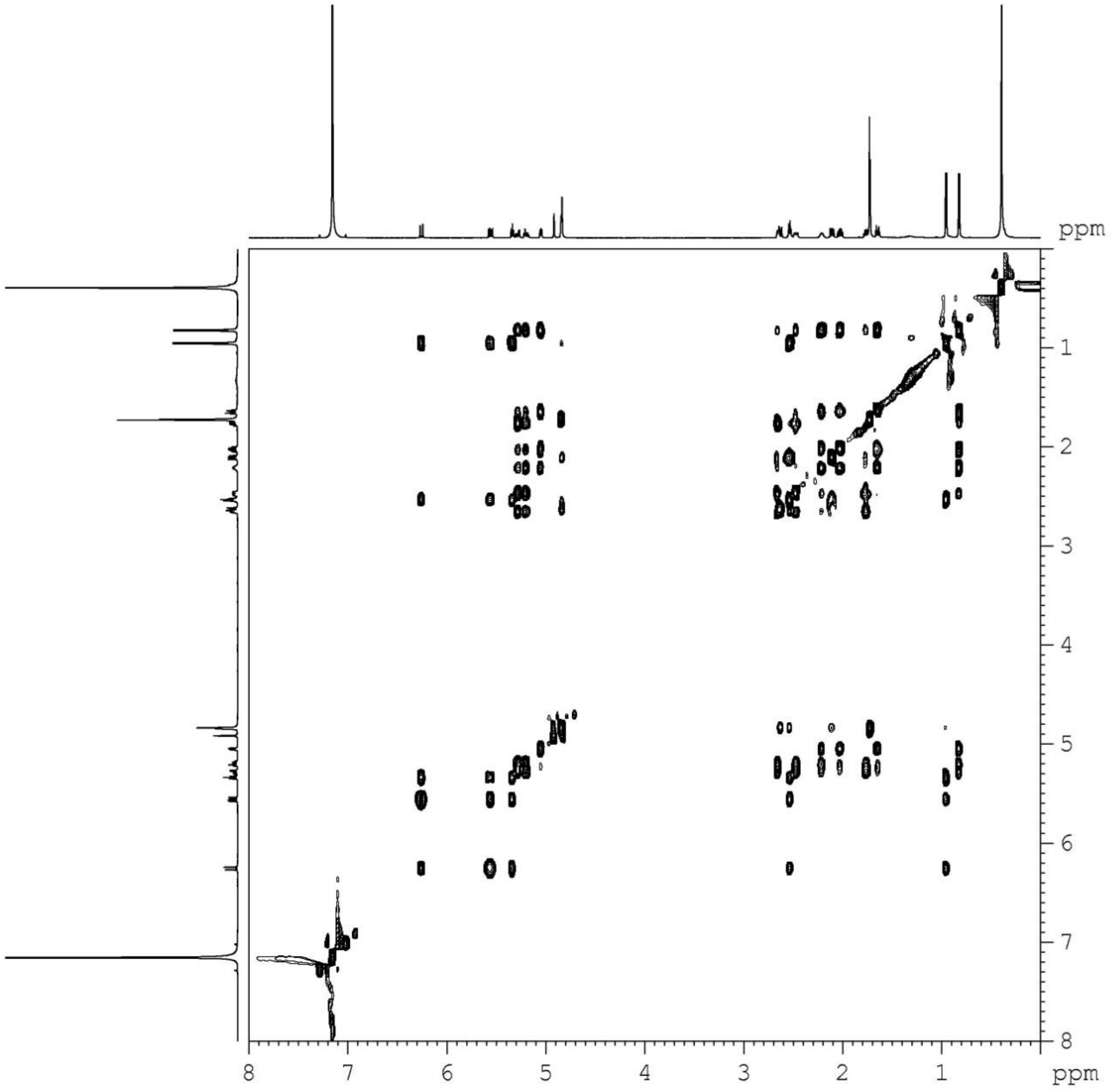

Figure S9 HSQCed NMR spectrum of **2** (600 MHz, C_6D_6)

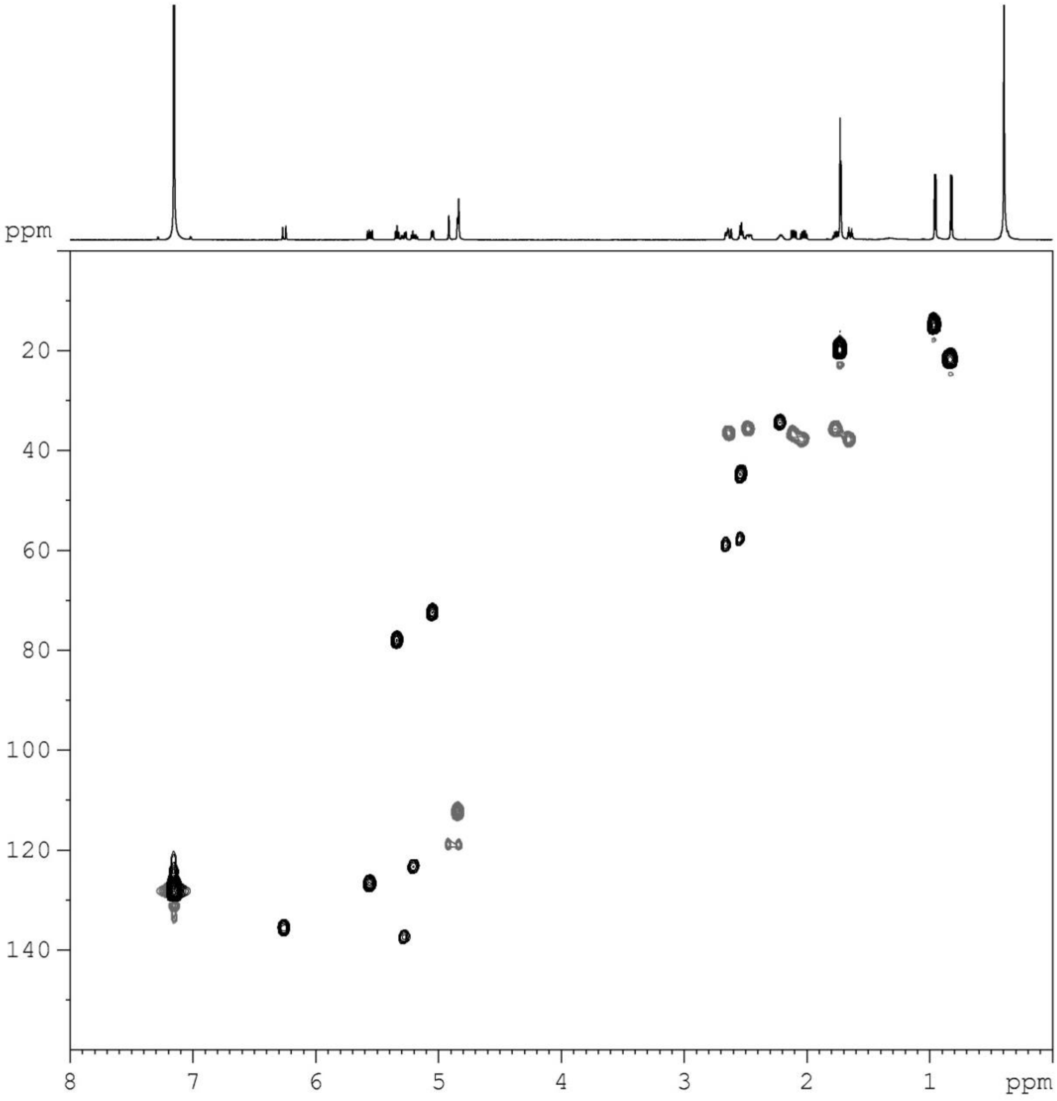

Figure S10 HMBC NMR spectrum of **2** (600 MHz, C_6D_6)

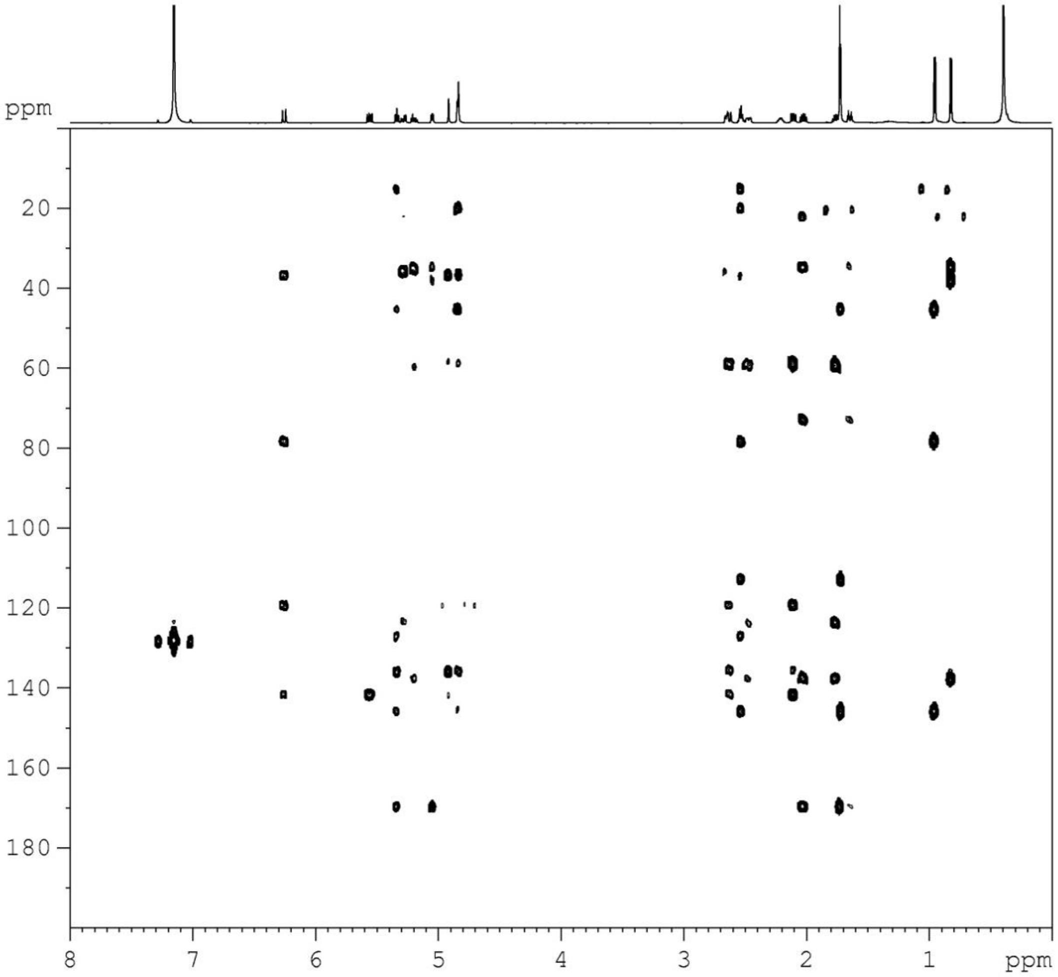

Figure S11 NOESY NMR spectrum of **2** (600 MHz, C_6D_6)

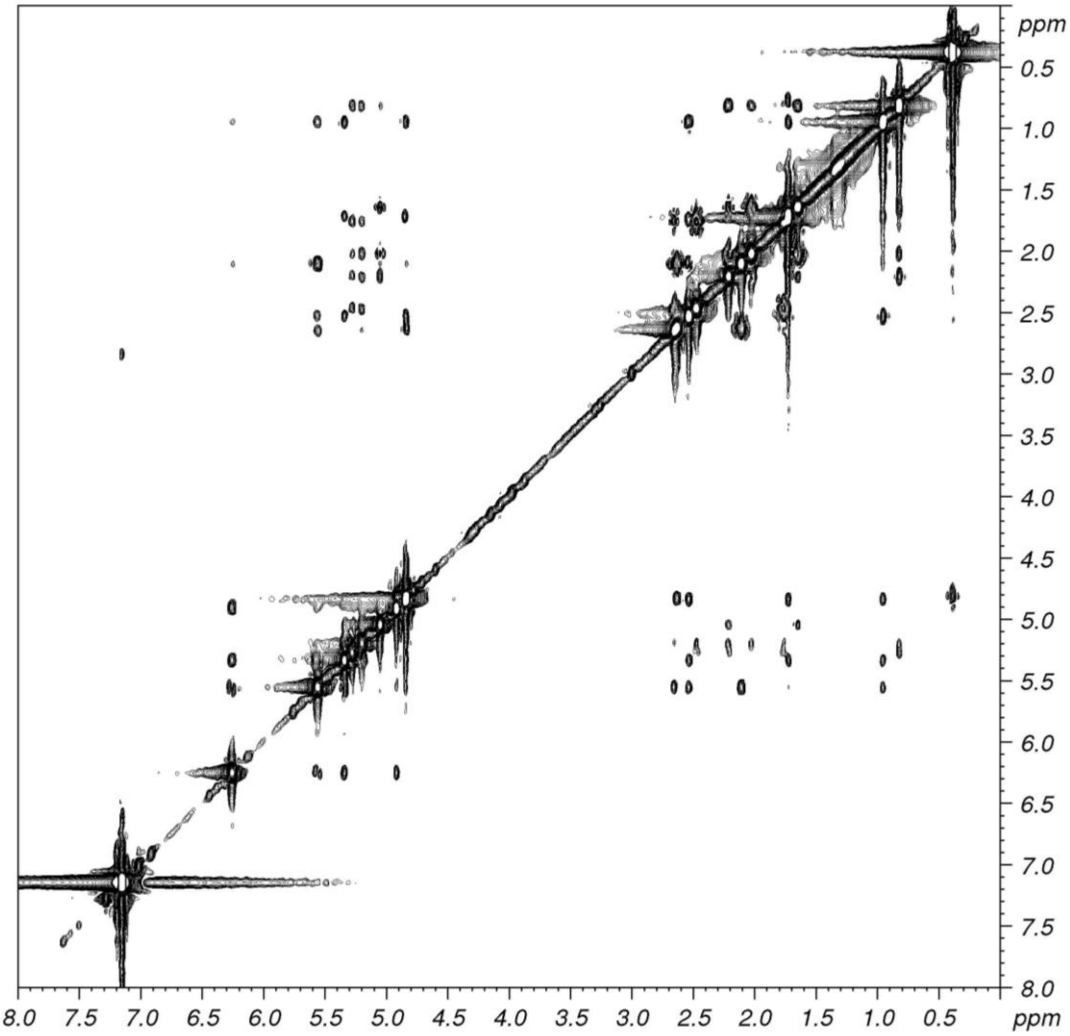

Figure S12 ^{1}H NMR spectrum of **2** (600 MHz, $CDCl_3$)

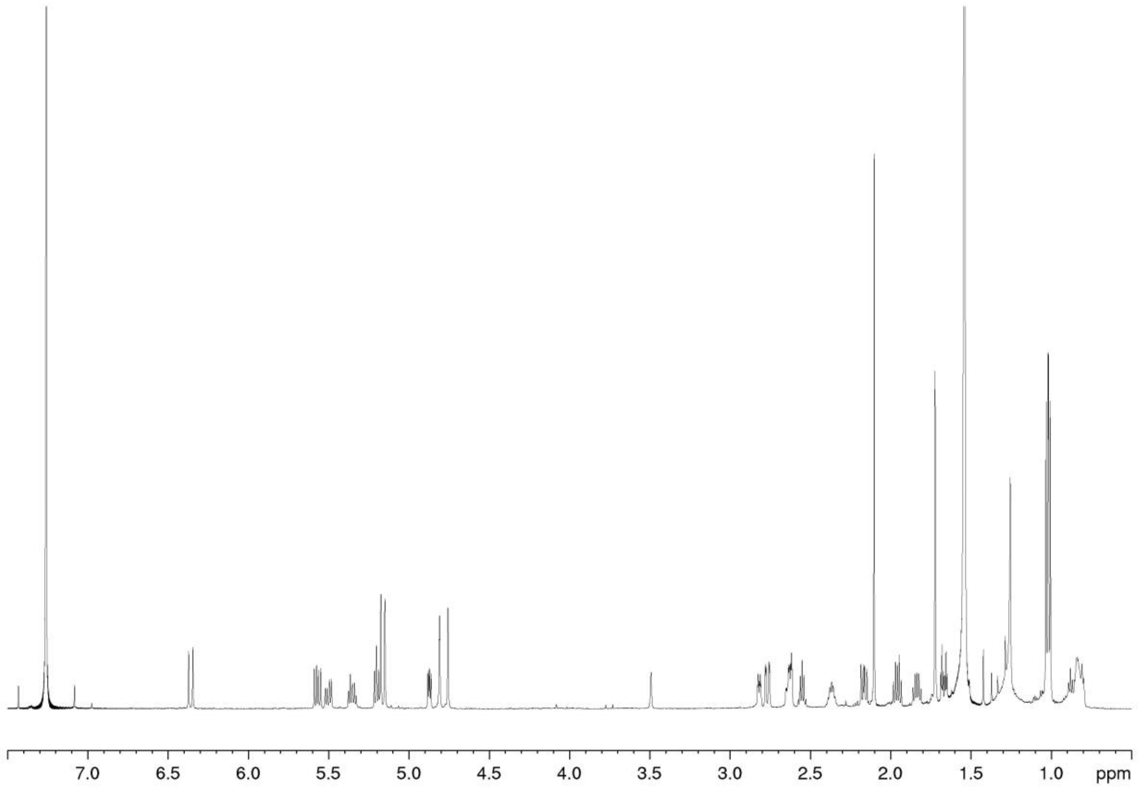

Figure S13 COSY spectrum of **2** (600 MHz, $CDCl_3$)

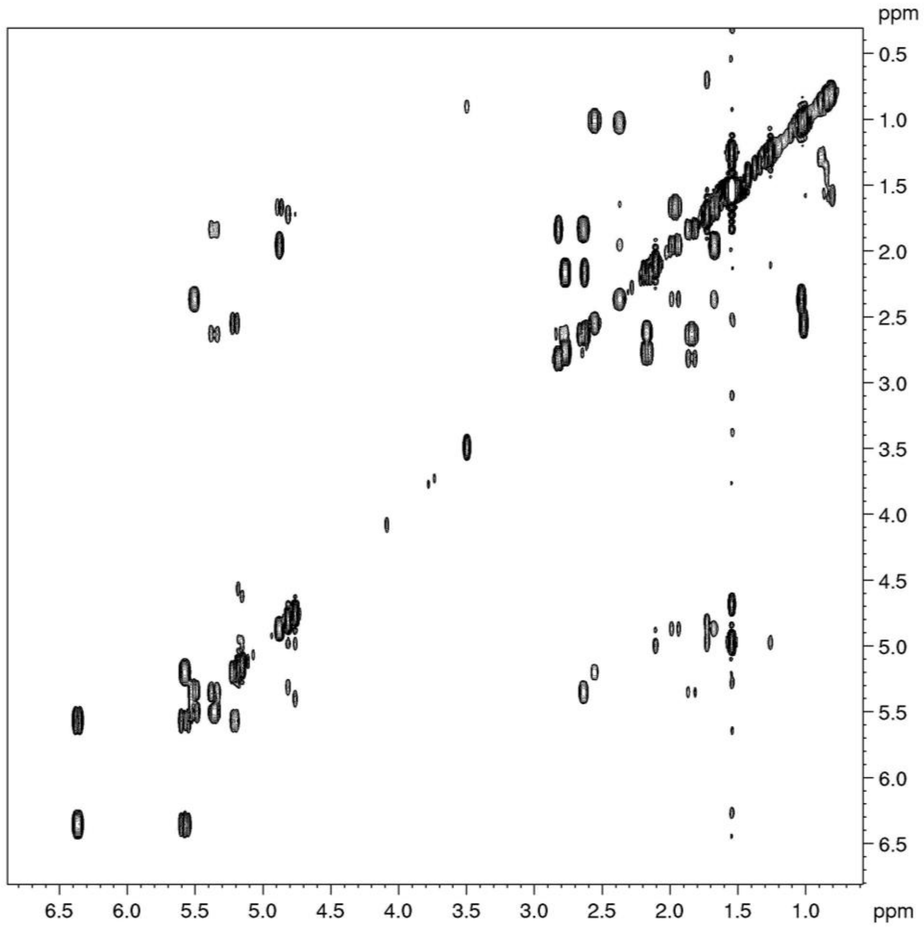

Figure S14 HSQCed spectrum of **2** (600 MHz, $CDCl_3$)

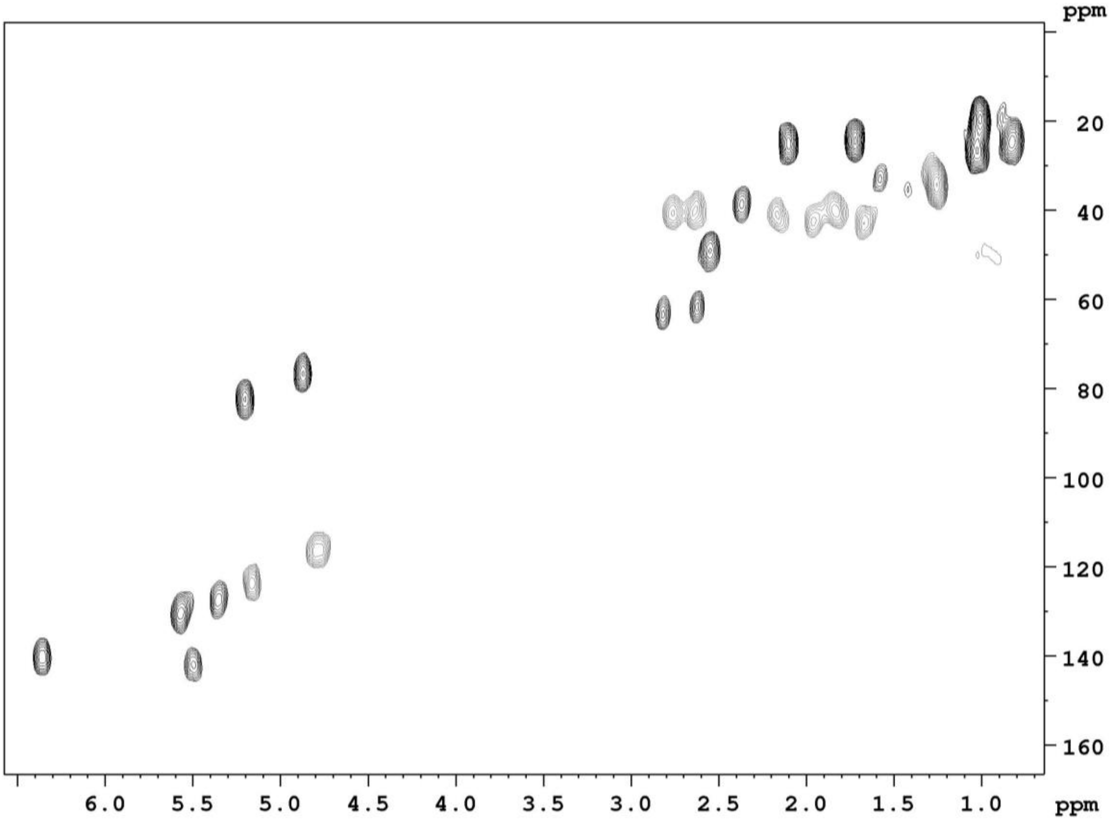

Figure S15 HMBC spectrum of **2** (600 MHz, $CDCl_3$)

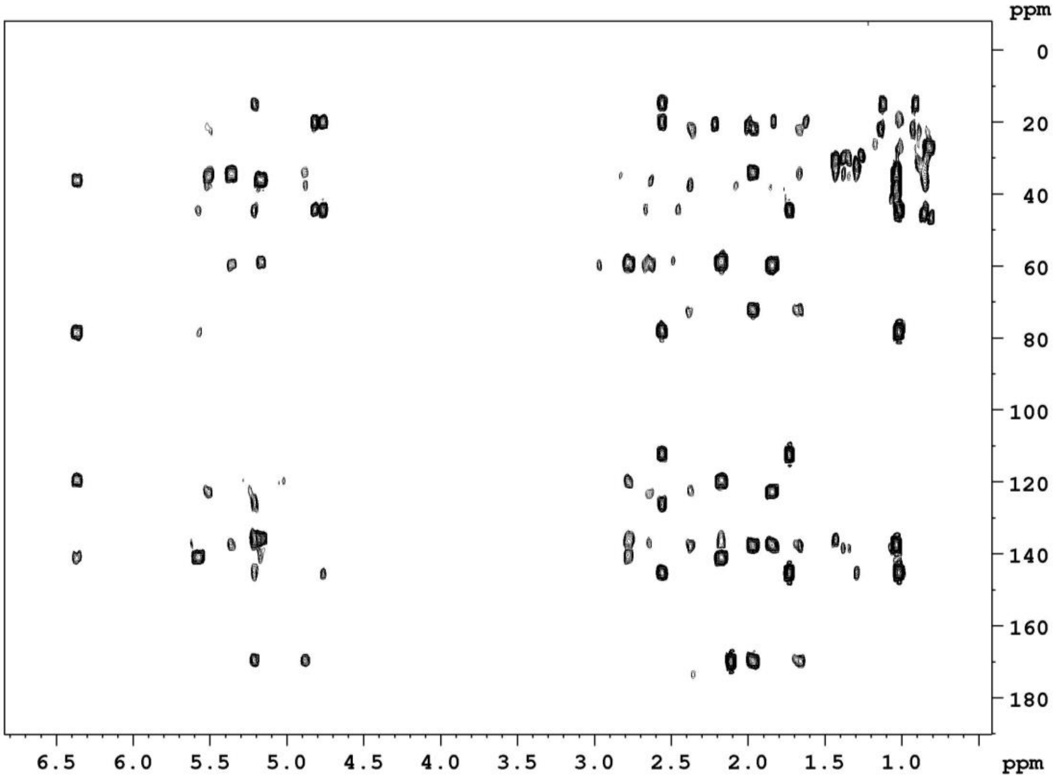

Figure S16 NOESY spectrum of **2** (600 MHz, $CDCl_3$)

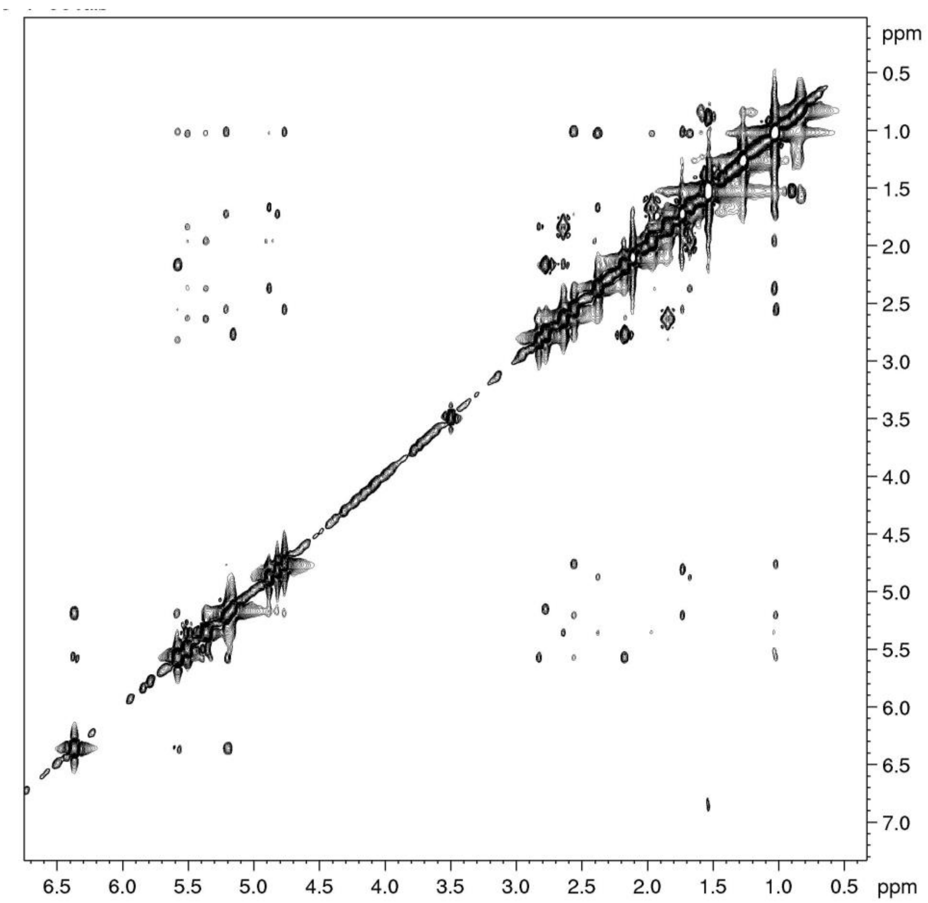

Figure S17 ^{1}H NMR spectrum of **3** (600 MHz, C_6D_6)

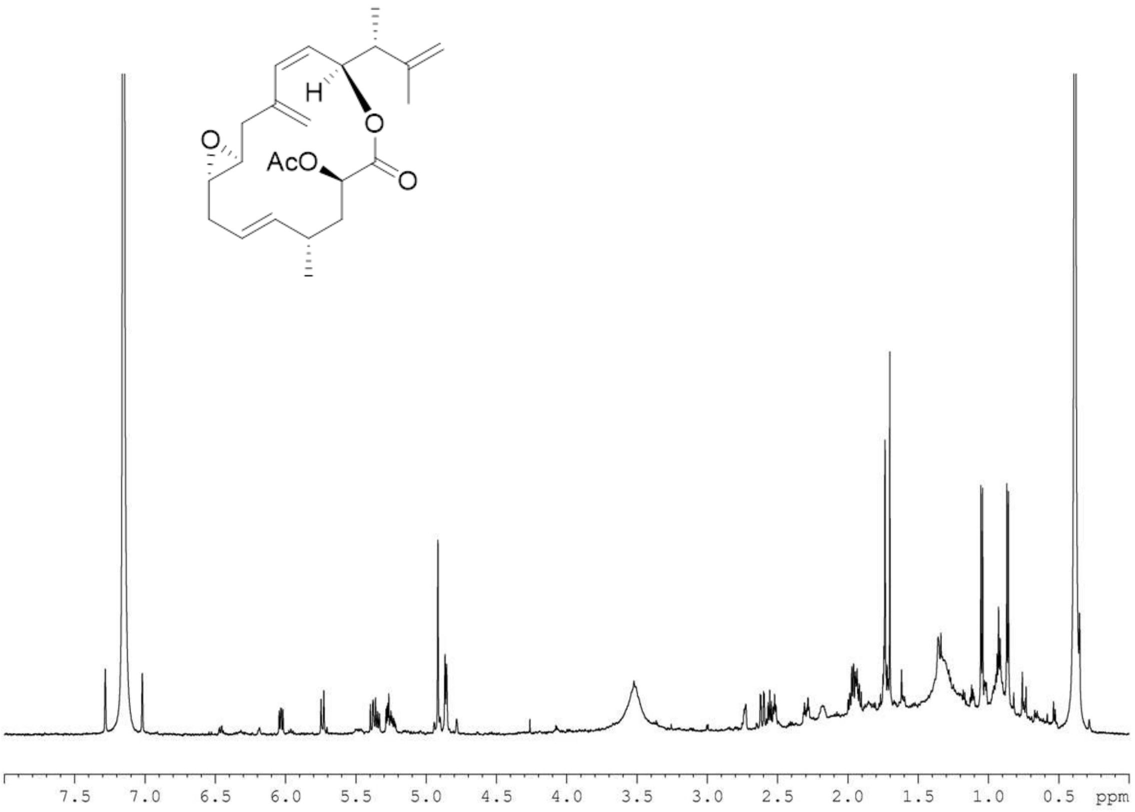

Figure S18 ^{1}H homo-decoupling spectrum of **3** (600 MHz, C_6D_6)

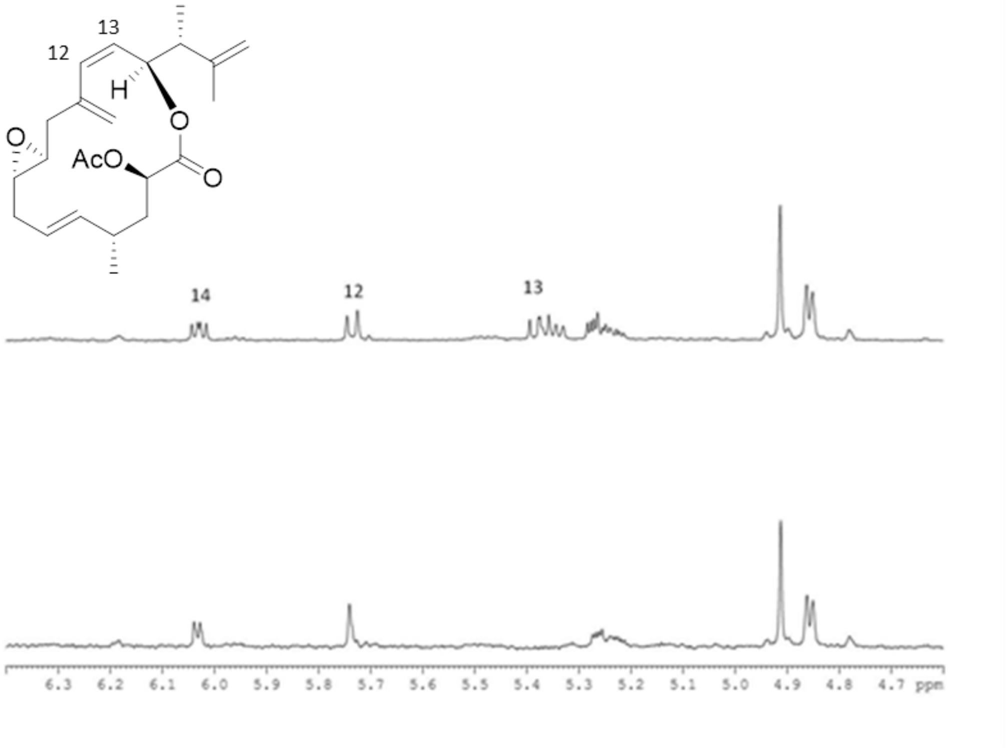

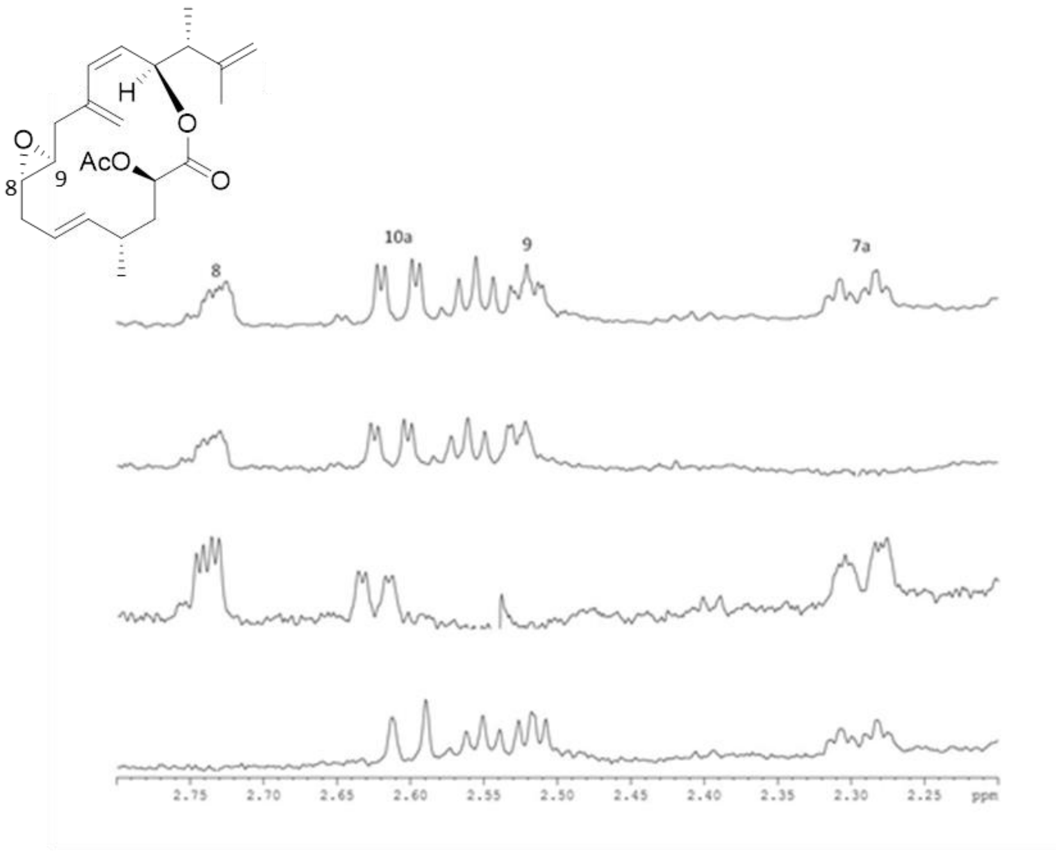

Figure S19 COSY NMR spectrum of **3** (600 MHz, C_6D_6)

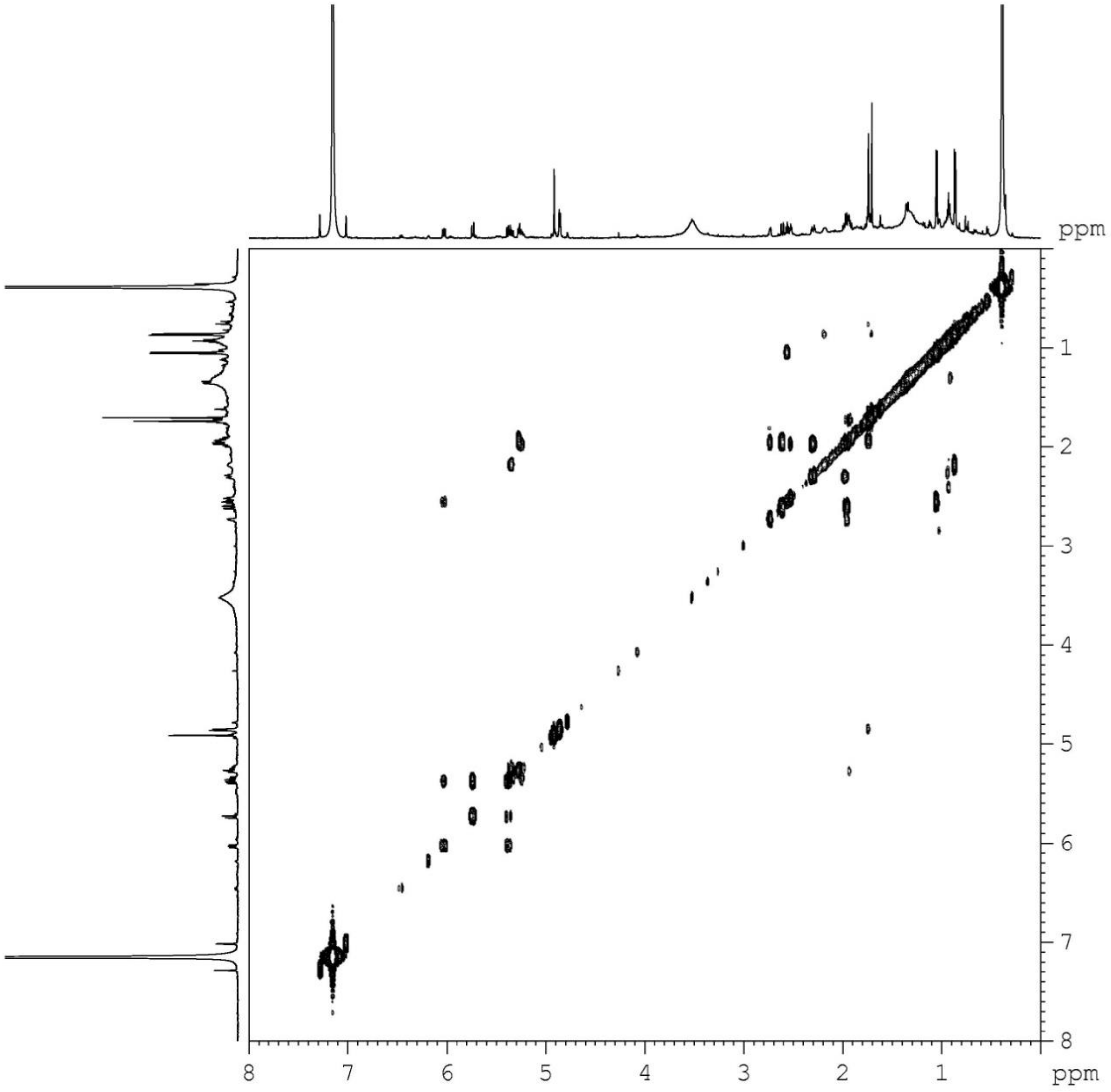

Figure S20 TOCSY NMR spectrum of **3** (600 MHz, C_6D_6)

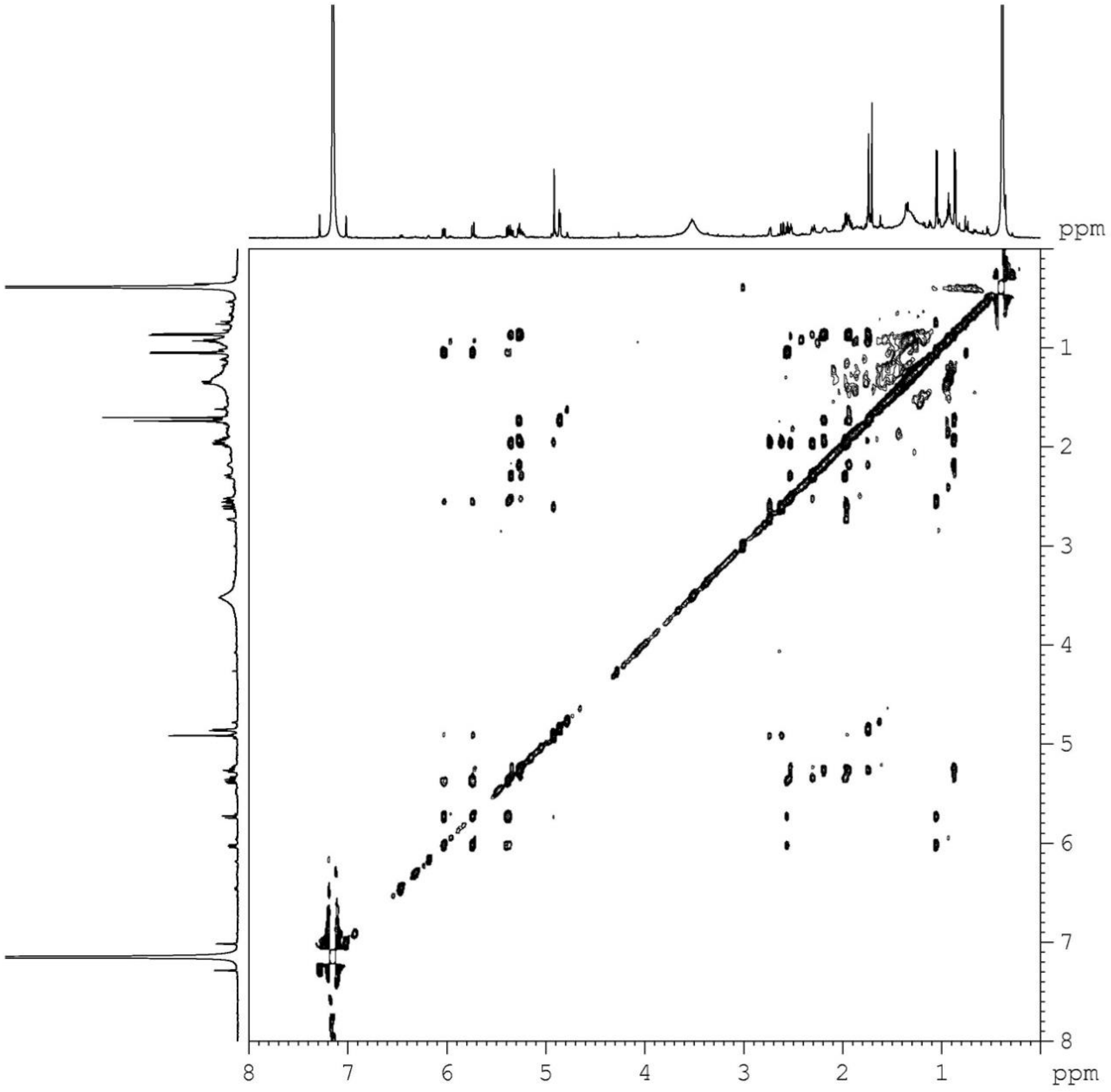

Figure S21 ^{1}H NMR spectrum of **3** (600 MHz, $CDCl_3$)

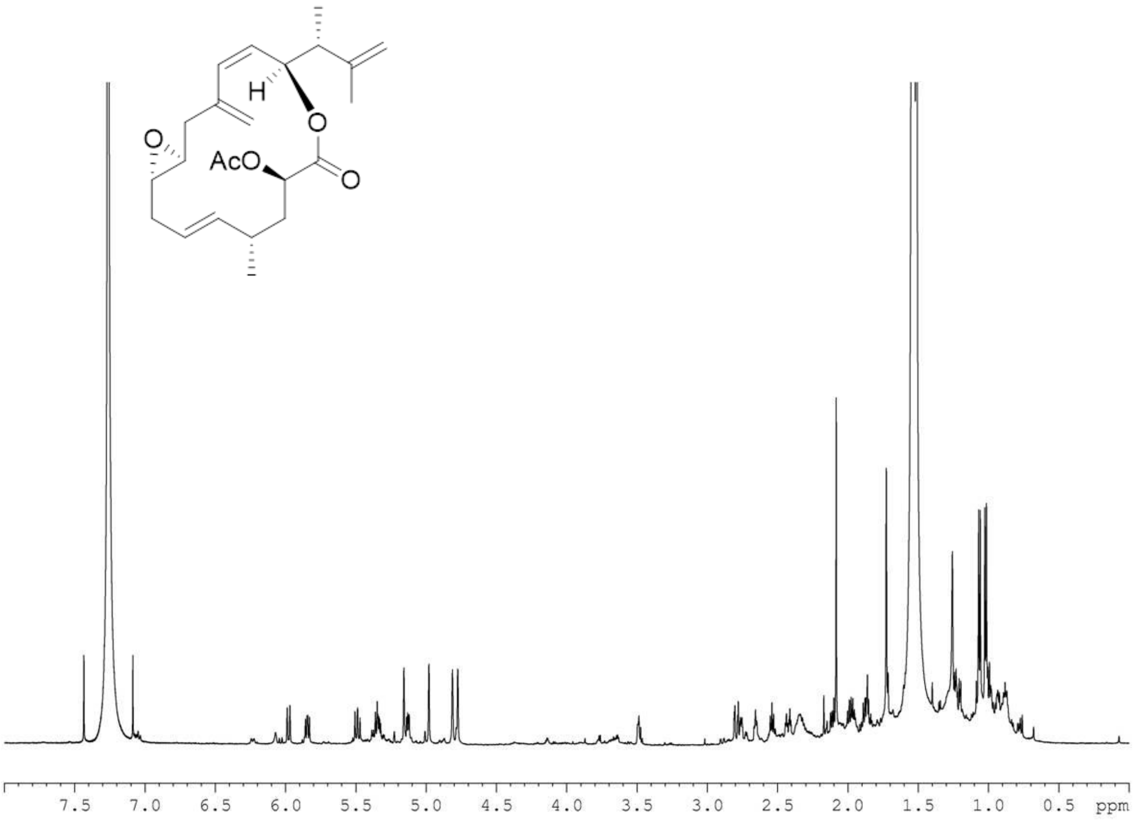

Figure S22 COSY NMR spectrum of **3** (600 MHz, $CDCl_3$)

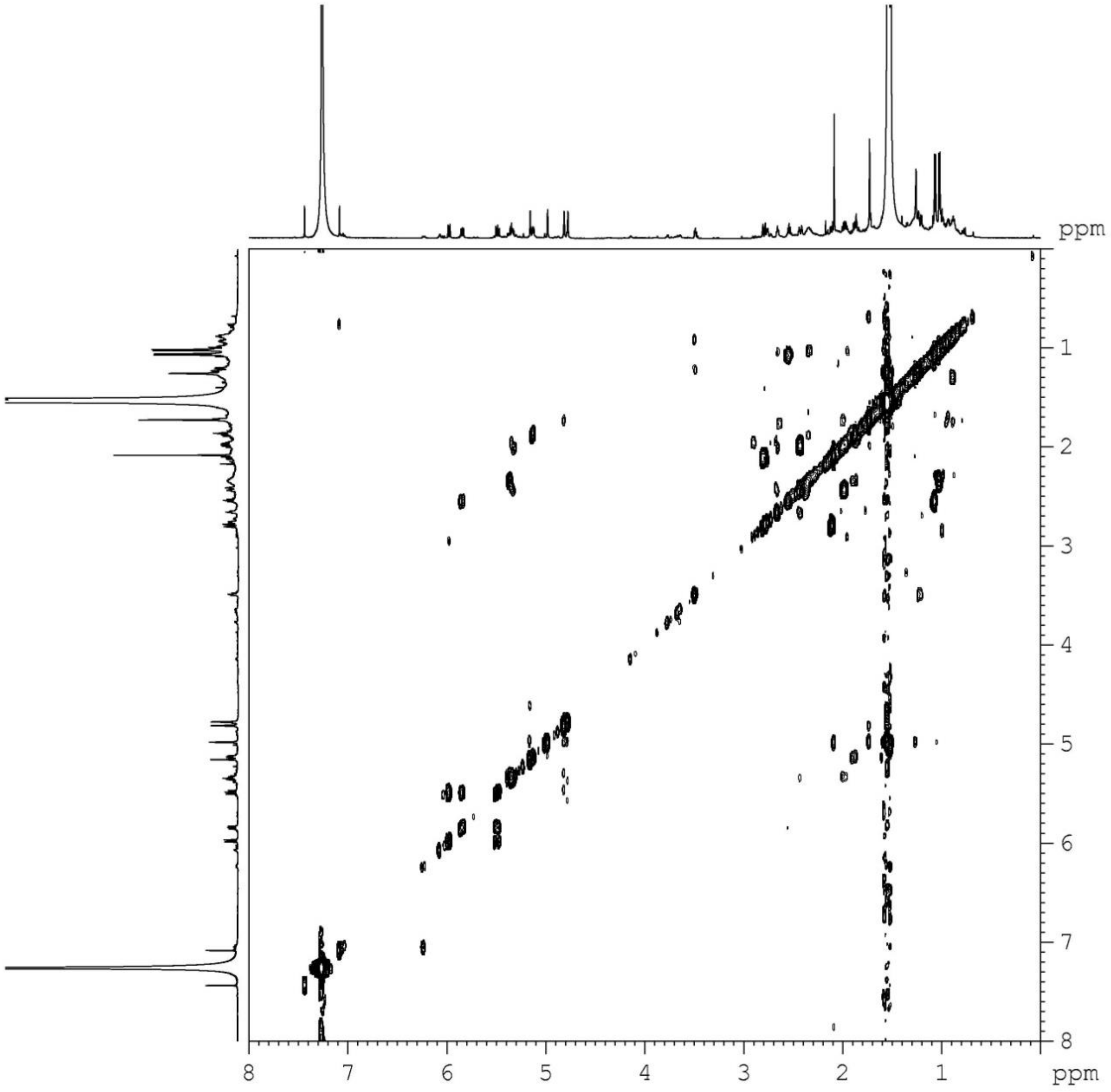

Figure S23 TOCSY NMR spectrum of **3** (600 MHz, $CDCl_3$)

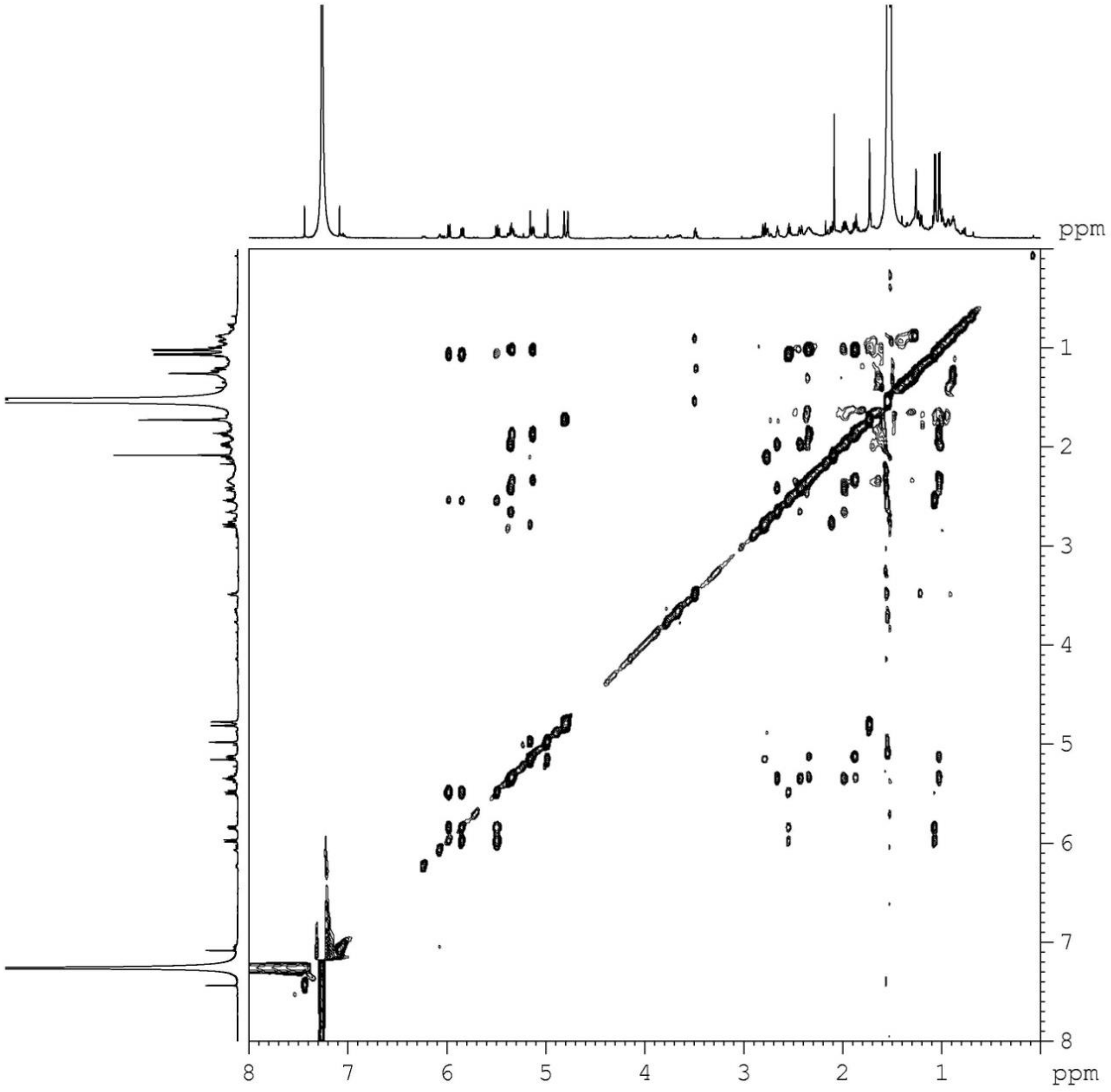

Figure S24 HSQCed NMR spectrum of **3** (600 MHz, $CDCl_3$)

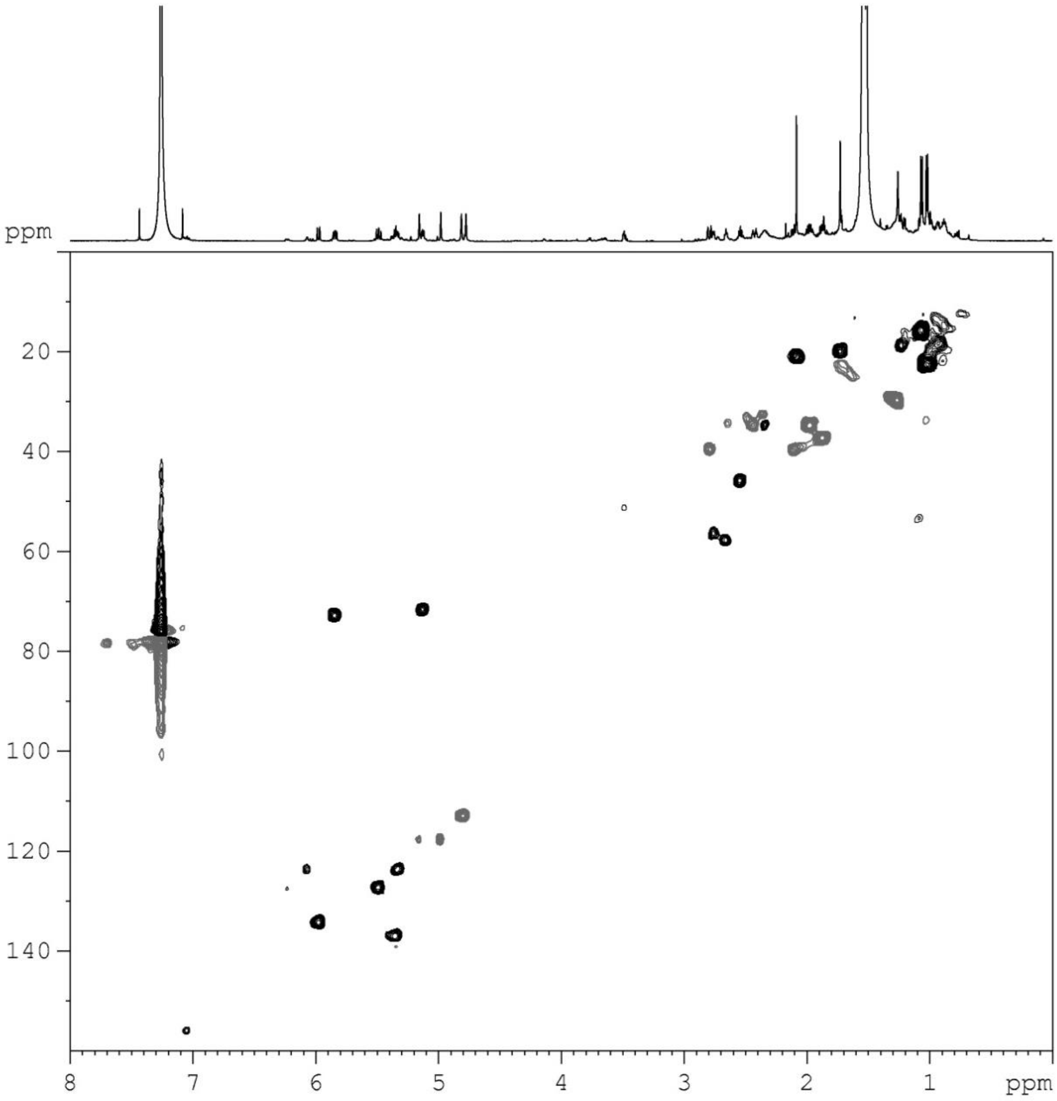

Figure S25 HMBC NMR spectrum of **3** (600 MHz, $CDCl_3$)

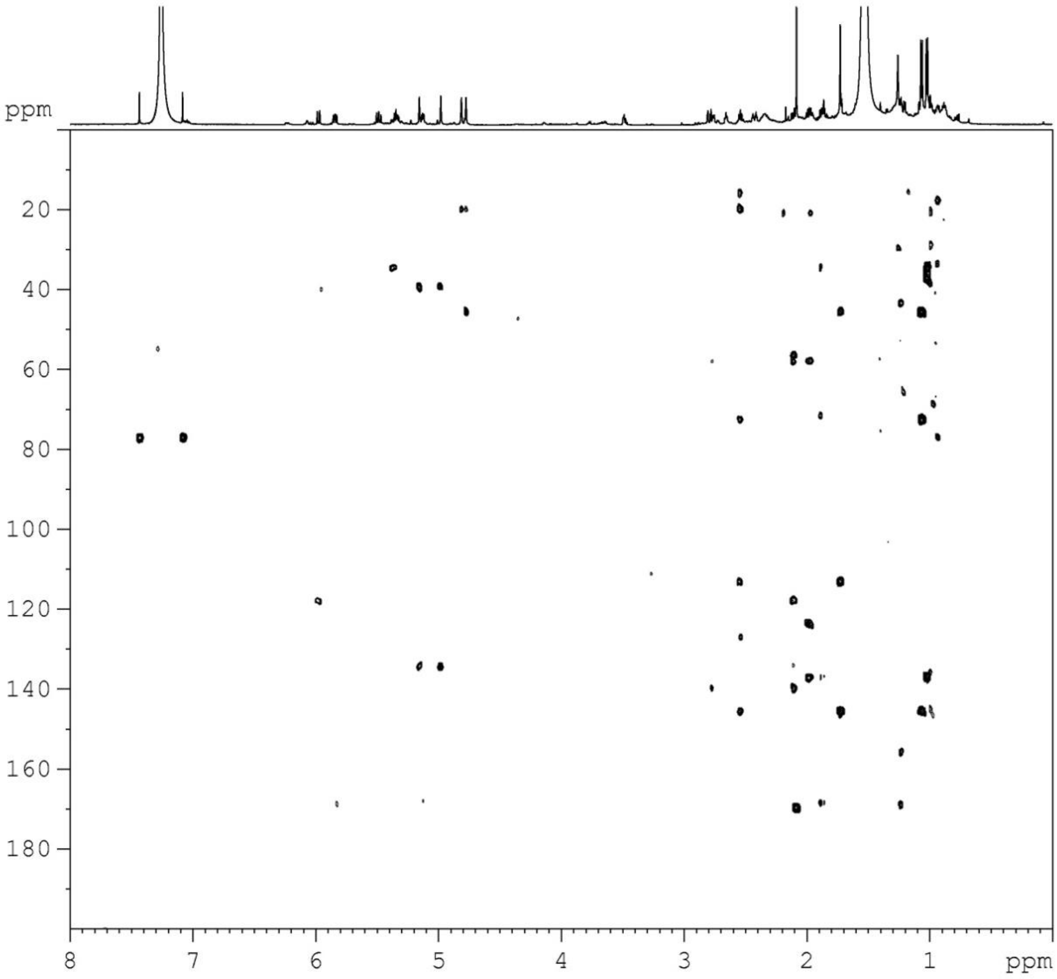

Figure S26 ^{1}H NMR spectrum of **4** (600 MHz, C_6D_6)

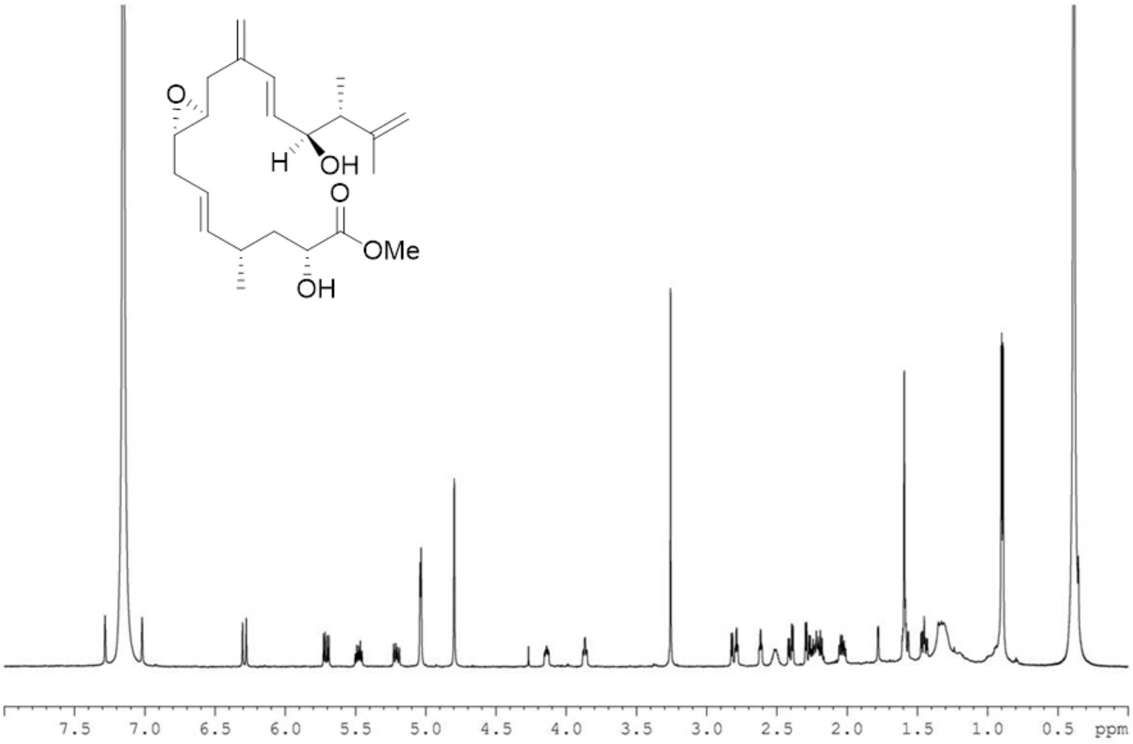

Figure S27 ^{1}H homo-decoupling spectrum of **4** (600 MHz, C_6D_6)

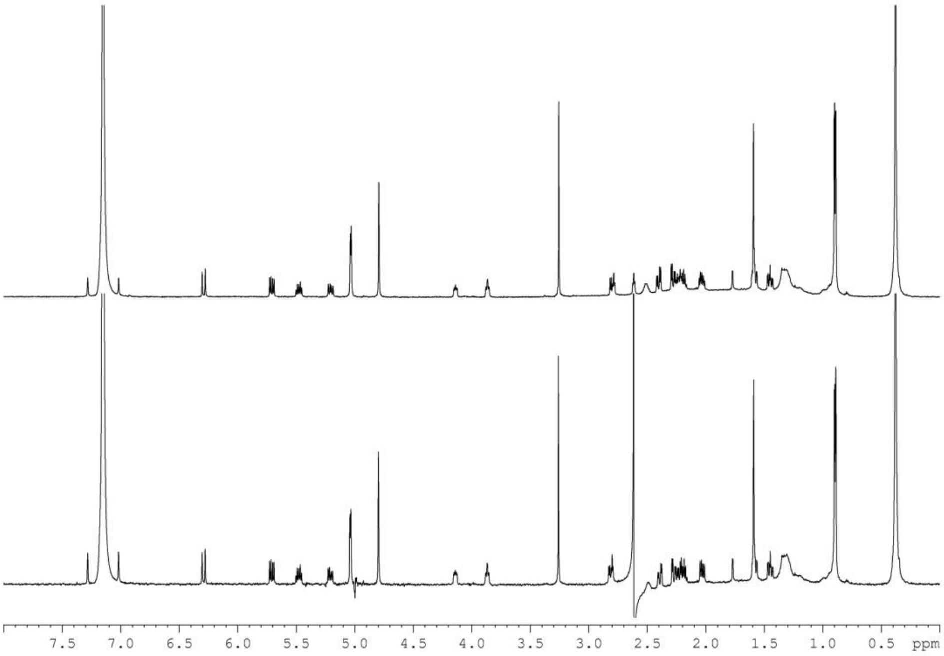

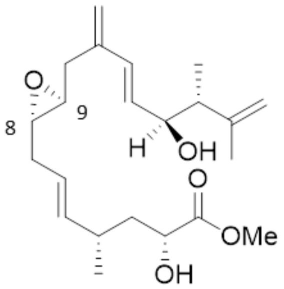

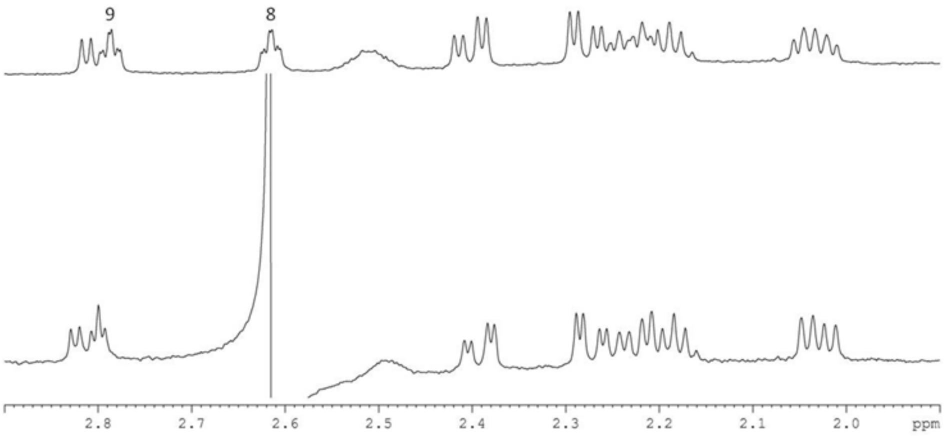

Figure S28 COSY NMR spectrum of **4** (600 MHz, C_6D_6)

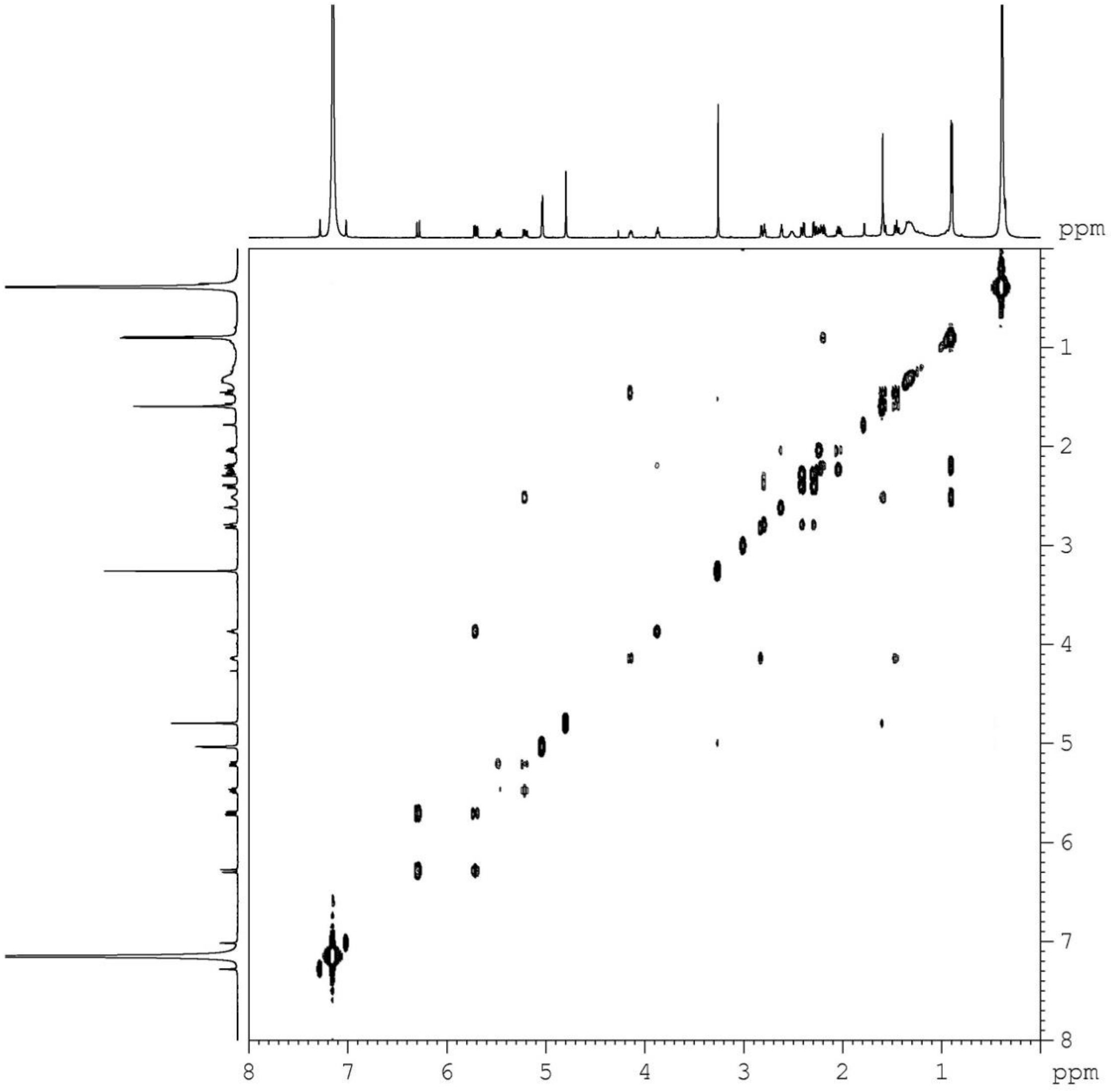

Figure S29 TOCSY NMR spectrum of **4** (600 MHz, C_6D_6)

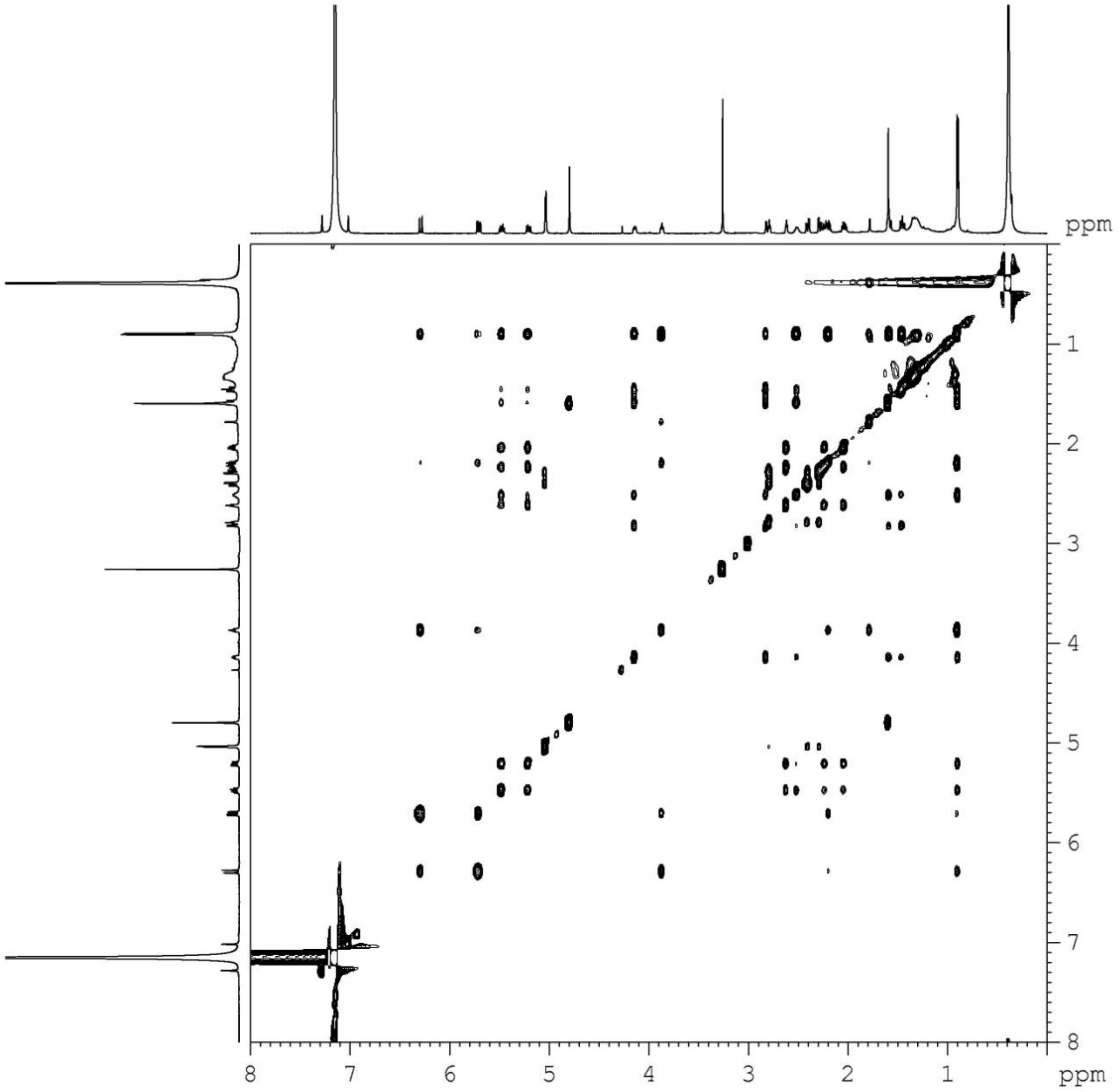

Figure S30 HSQCed NMR spectrum of **4** (600 MHz, C_6D_6)

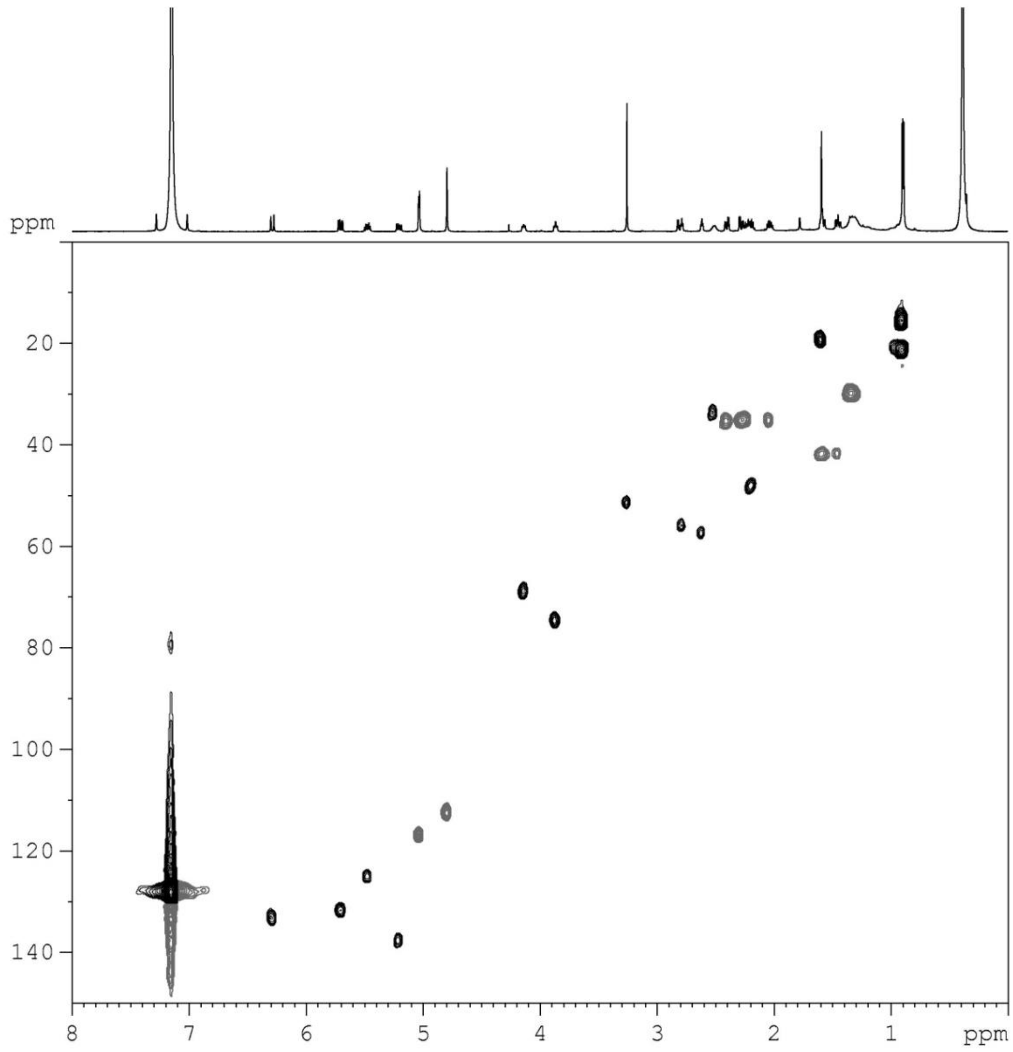

Figure S31 HMBC NMR spectrum of **4** (600 MHz, C_6D_6)

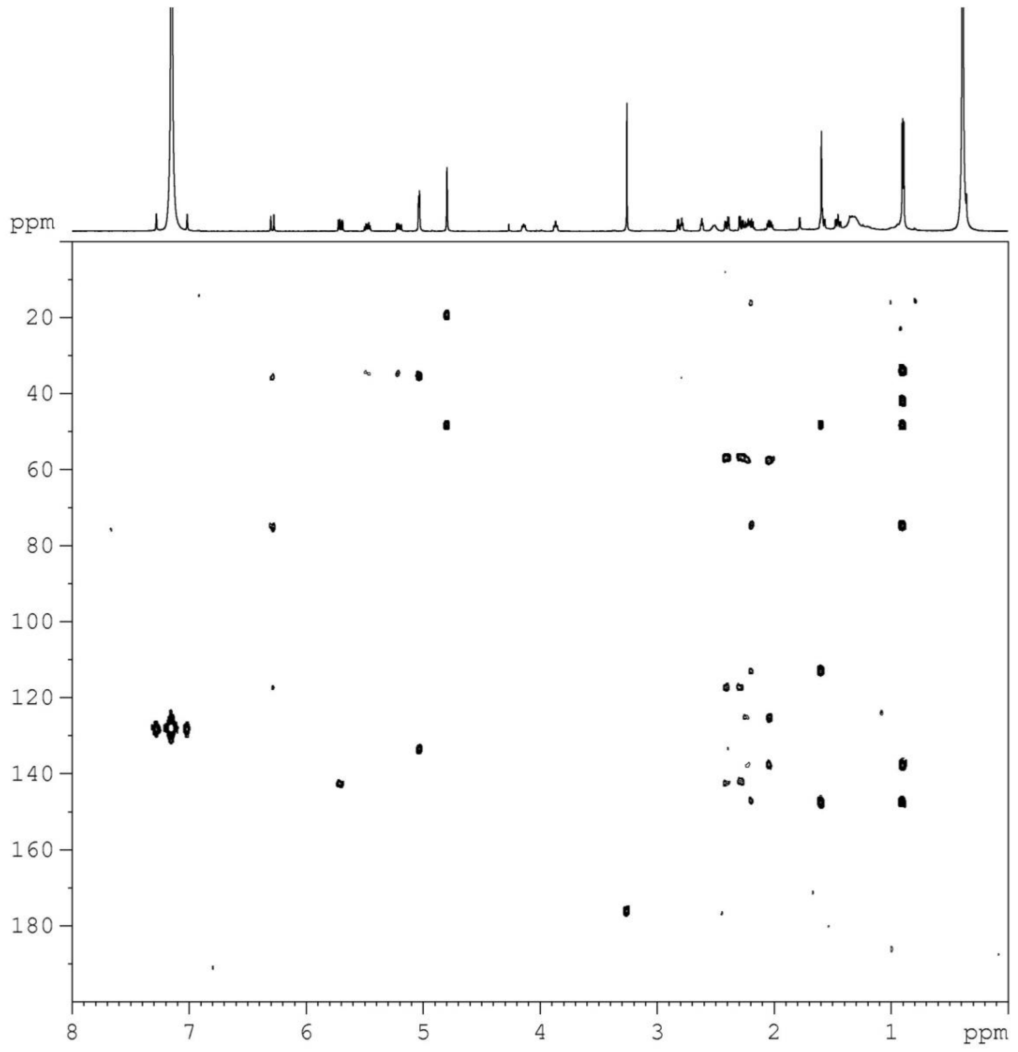

Figure S32 ^1H NMR spectrum of **4a** (600 MHz, $CDCl_3$)

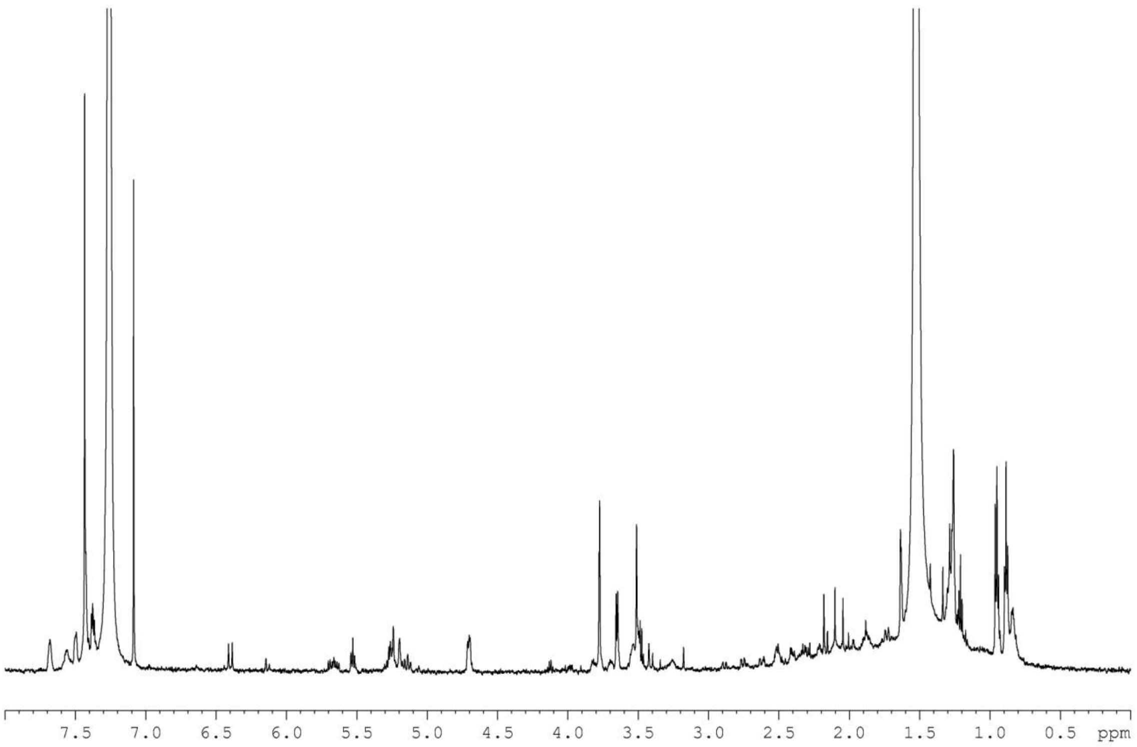

Figure S33 COSY NMR spectrum of **4a** (600 MHz, $CDCl_3$)

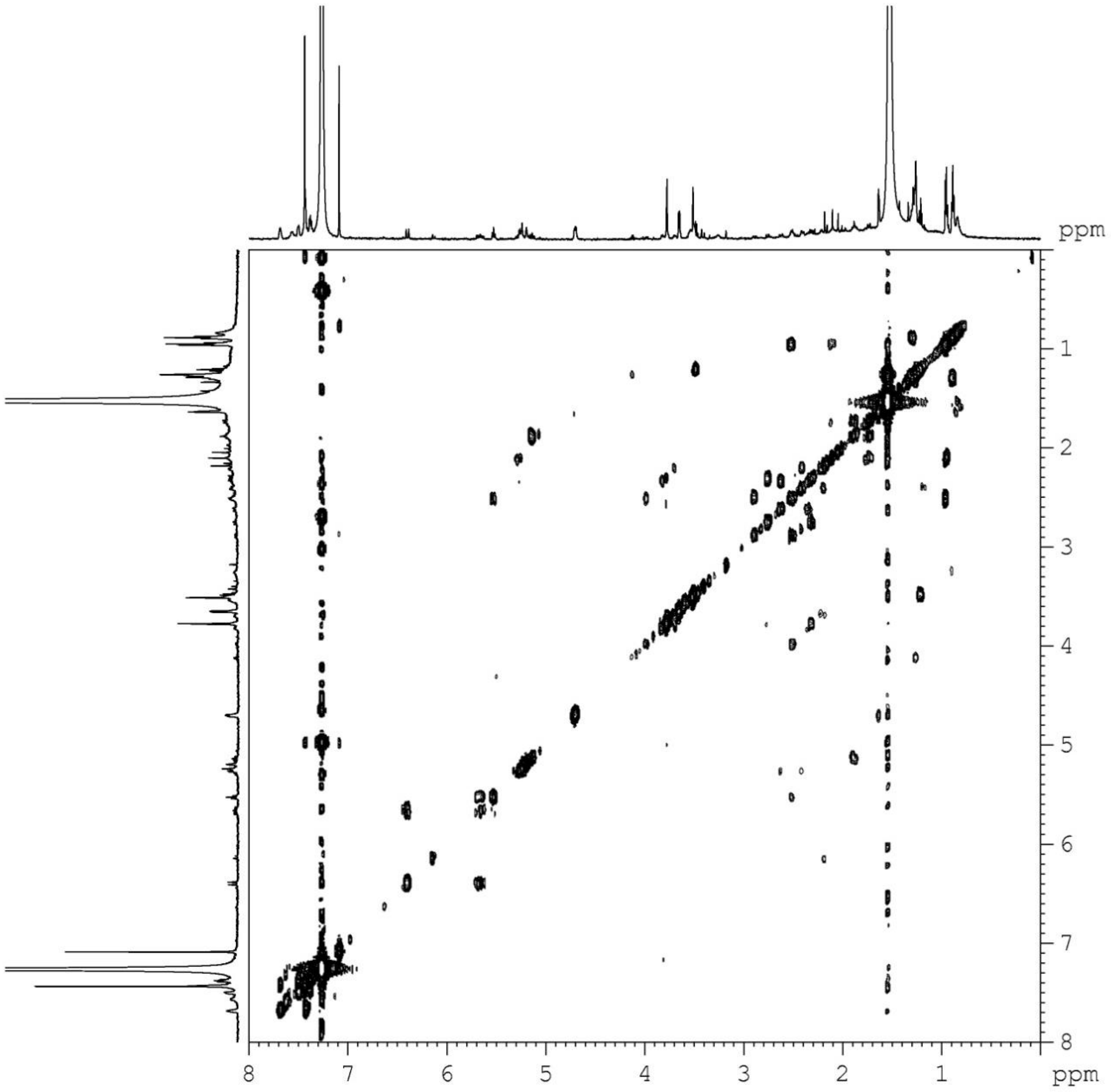

Figure S34 TOCSY NMR spectrum of **4a** (600 MHz, $CDCl_3$)

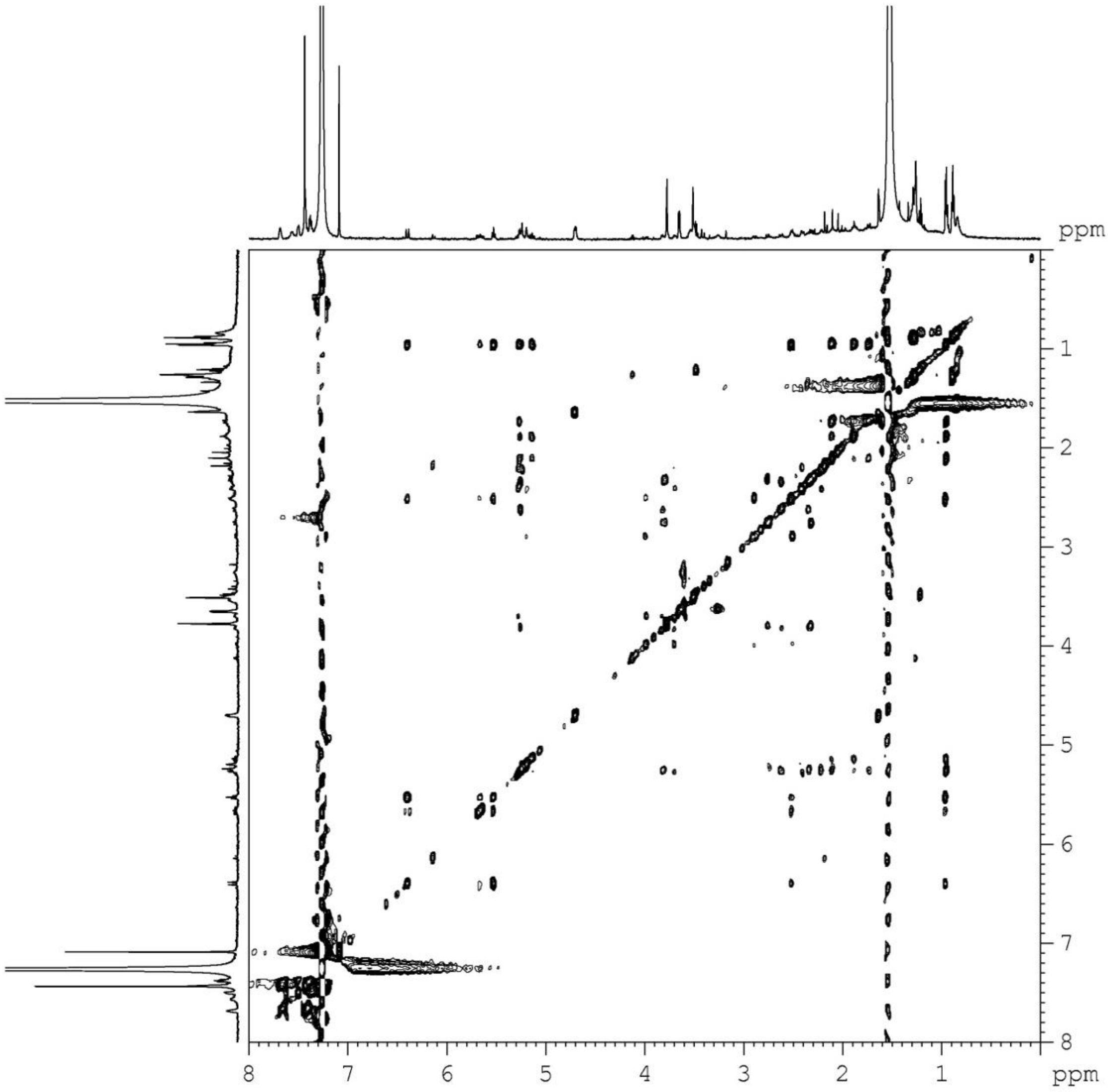

Figure S35 ^{1}H NMR spectrum of **4b** (600 MHz, $CDCl_3$)

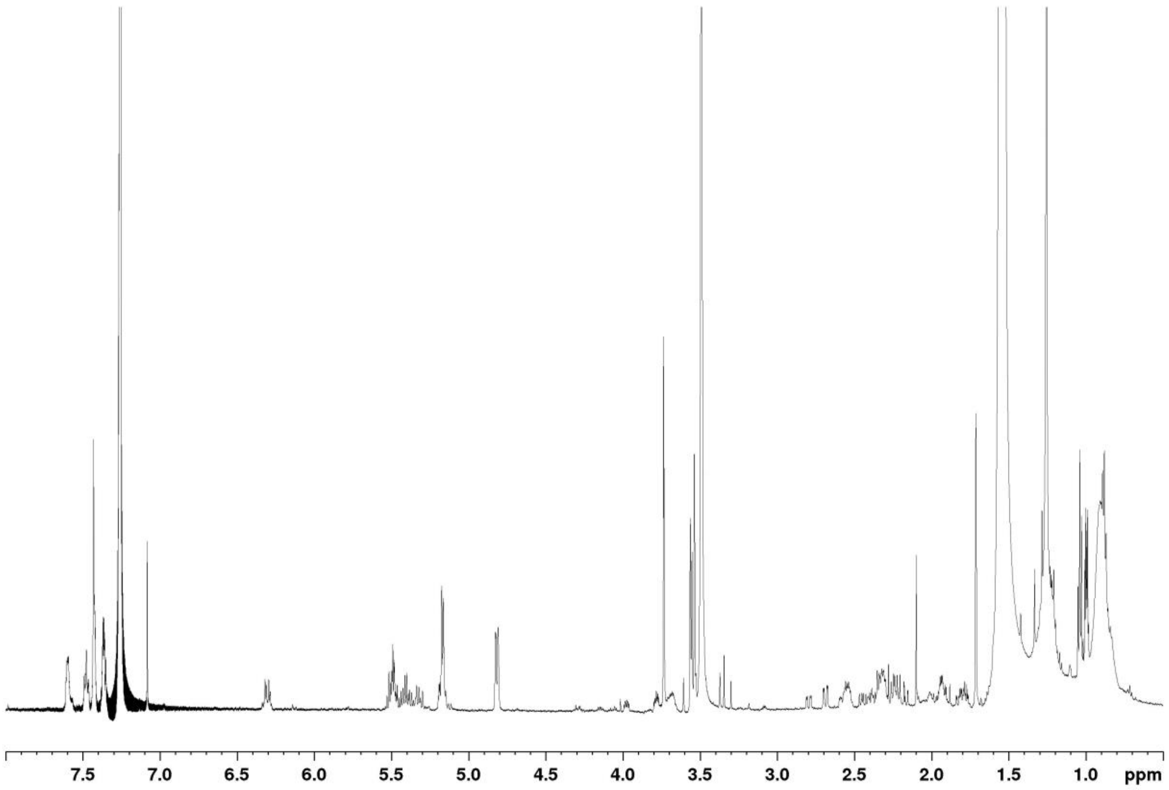

Figure S36 COSY NMR spectrum of **4b** (600 MHz, $CDCl_3$)

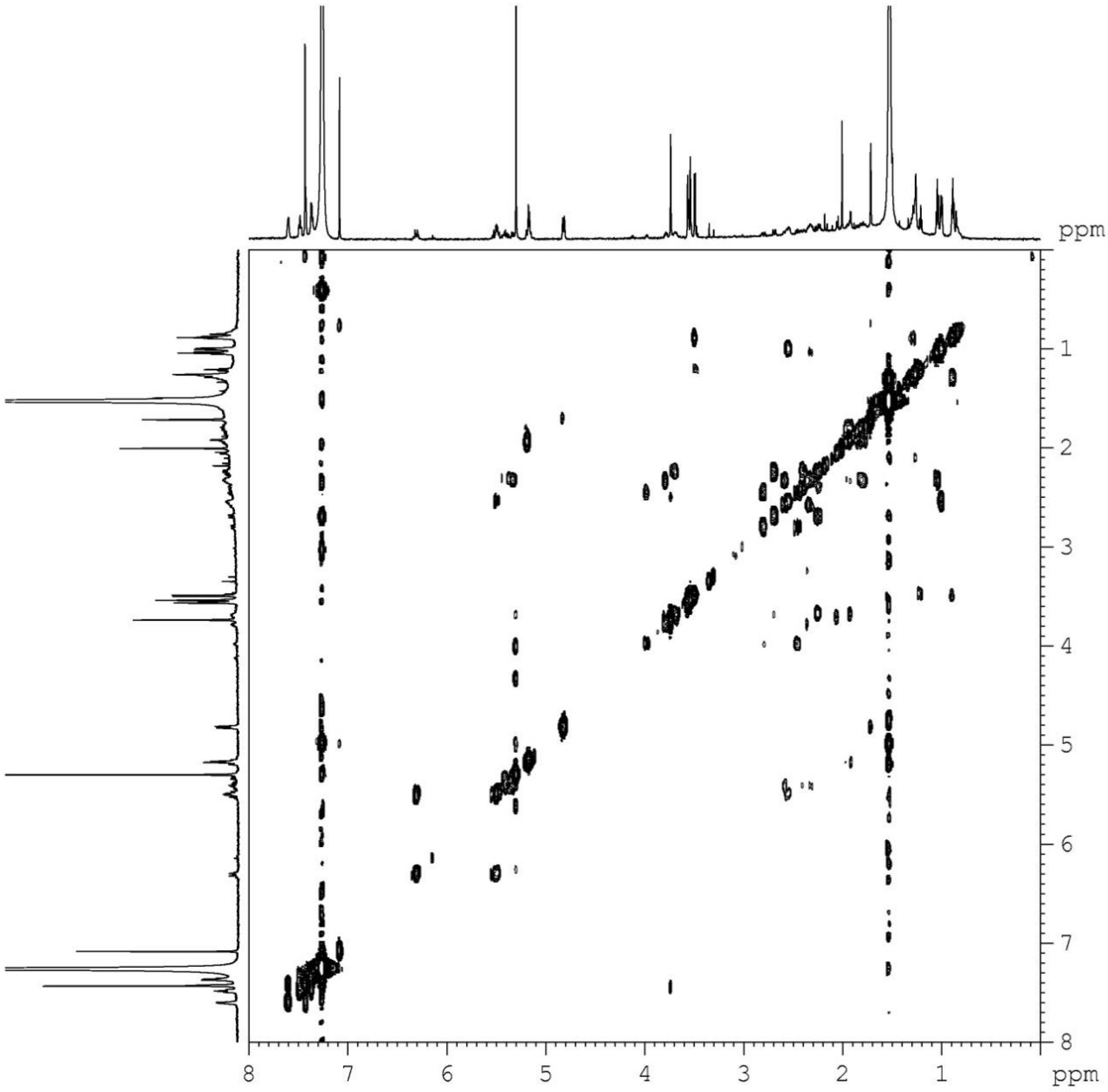

Figure S37 TOCSY NMR spectrum of **4b** (600 MHz, $CDCl_3$)

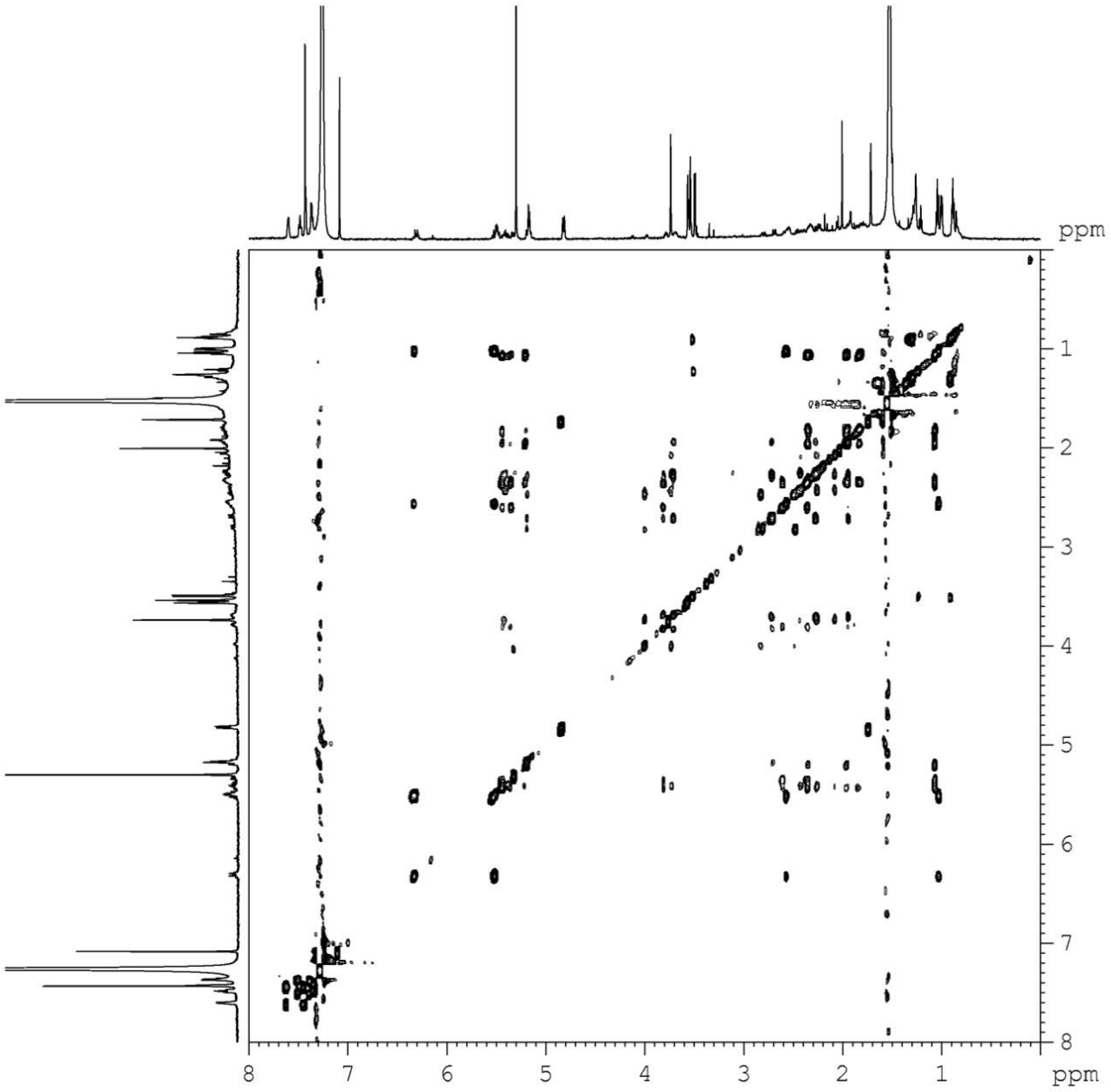

Figure S38 NOESY (double arrow) correlation of **2**

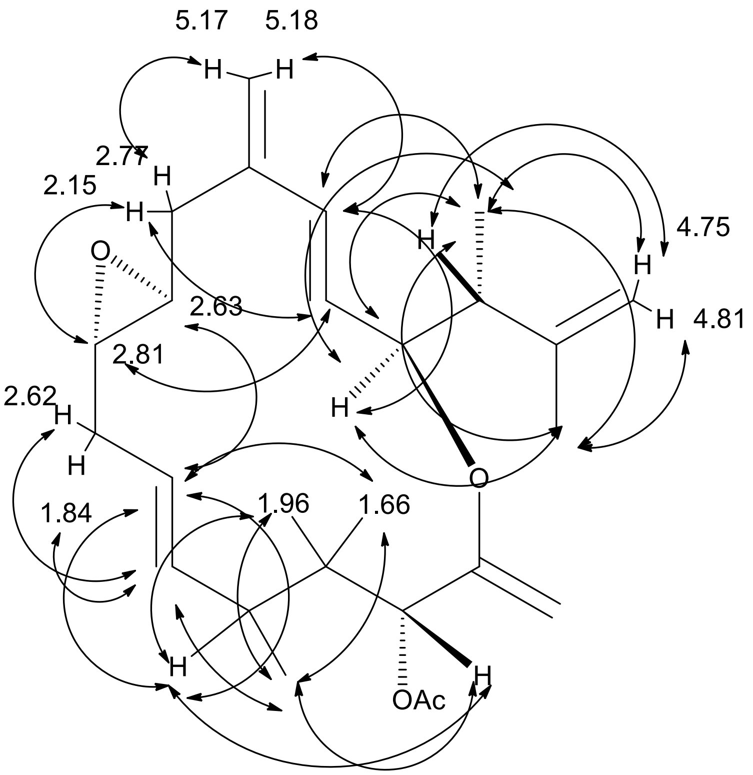

Refinement protocol of Simulated Annealing (SA), Molecular Dynamics (RM) and Energy Minimization (EM).

Simulations on compound **2** were run with Amber 17 package [1] by using the version 2 of GAFF force field [2] with charges derived at MP2/6-31G* level with the RESP procedure [2]. GAMESS program (version 30 Sept. 2017, R2) [2,3] was employed for quantum chemistry calculations. An integration timestep of 2 fs was used both in SA and in pure MD runs, performed with a Langevin dynamics approach to regulate temperature (collision frequency = 10 ps-1) [3,4]. The length of bonds involving hydrogen atoms was constrained with the SHAKE procedure [5]. The Analytical Linearized Poisson-Boltzmann (ALPB) implicit solvation [6,7] with GB*n* model parameters [8] was used in all SA and MD calculations to simulate the presence of the solvent used in NMR spectra from which the experimental information was derived (benzene). The effective electrostatic radius of the molecule required by ALPB method was calculated from 250 ps unrestrained MD simulations on both 4*R* and 4*S* diastereomers and then averaged (final value = 4.998 Å). Restraints were used to ensure preservation of the proper chirality of stereocenters (by restraining the corresponding improper dihedrals centered on the stereocenter) and the cis/trans conformation of double bonds (by restraining the corresponding dihedral angle). A "r" prefix indicates the use of restraints in SA, MD or EM calculations. NMR information from NOE spectra was translated into either lower- or upper-plus lower-limit distance restraints, represented by flat-bottomed half-parabolic functions switching to a linear form, for "antiNOEs" and "NOEs" respectively. AntiNOEs are cross-peaks between couples of protons that are surely missing in the NOESY spectra (i.e. not potentially covered by noise, solvent, other cross-peaks). In particular, a half-parabolic penalty function with a minimum (corresponding to zero penalty) located at the upper/lower limit is applied on the right/left side of the minimum with force constant K_d, and this function is switched after 0.5 Å to a linear form ensuring continuity to the function and its derivative. Maximum values of K_d^{max}=20 kcal mol^{-1} Å-2 for distances and K_φ^{max}=20 kcal mol^{-1} rad^{-2} for dihedral angles were used. The following time profiles for temperature (T) and distance restraint K_d were applied in the rSA+rMD protocol (angle restraints were always fully operative at K_φ^{max}): from t=0 to 0.2 ns T is linearly increased from 300 to 1200 K, from t=0.2 to 1.0 ns T is kept fixed at 1200 K, from t=1.0 to 2.0 ns T is linearly decreased from 1200 to 300 K, in the final MD from t=2.0 to 3.0 ns T is kept fixed at 300 K; from t=0 to 0.4 ns the SA is run fully unrestrained (K_d=0.0), from t=0.4 to t=0.8 ns K_d is linearly increased from 0.0 to K_d^{max}, from t=0.8 ns to the end (3.0 ns) K_d is kept fixed at K_d^{max}. AntiNOEs were translated into 3.2 Å lower-limit restraints (except for restraint featuring one methyl group, for which a 3.7 Å lower-limit restraint distance was used), thus allowing the identification of negative violations in conformer ensemble averages whenever the contribution of a conformer with short (<2.5 Å) to medium (<3.0 Å) distance is greater than about 5% to 10%, respectively. NOEs were translated into lower/upper-limit restraints by integrating the peaks of the spectrum in benzene determined with a mixing time of 150 ms and employing the proportion $V_p/V_{ref}=(r_{ref}/r_p)^6$, where V_p and V_{ref} are the volumes of the cross-peak to be translated into a restraint and of a reference peak corresponding to a fixed distance in the molecule, respectively, and rp/rref are the corresponding interprotonic distances. The cross-peak between H12 and H13 atoms was used as reference peak, with a corresponding r_{ref}=2.99 Å. The calculated distances were translated into lower/upper-limit ranges by subtracting/adding 0.3 Å. An extra 0.3 Å was added to the upper limit for each methyl group involved in the interaction. Whenever peaks in the 150 ms spectrum in benzene were too noisy or exhibited irregularities in their intensities in comparison with their corresponding counterparts in the 250 and 350 ms spectra, the corresponding peaks in the 350 ms spectrum in $CDCl_3$ were used. The final rMD simulation was run with the same setup described above for the MD run of the rSA+rMD+rEM protocol and the restraint force constant described in the text. Frames were saved every 0.1 ps of SA or MD, while RMSD clustering [9] was performed on MD structures sampled every 1 ps. Weighted conformer ensembles were generated by searching for the combination of conformer populations providing the least sum of weighted average restraint violations (WARV). WARVs are calculated with the following formula:

$WARV_{ij} = r_{ij}(ll) – r_{ij}(avg)$ $\quad (r_{ij}(avg) < r_{ij}(ll))$

$WARV_{ij} = 0$ $\quad (r_{ij}(ul) \le r_{ij}(avg) \le r_{ij}(ul))$

$WARV_{ij} = r_{ij}(avg) – r_{ij}(ul)$ $\quad (r_{ij}(avg) > r_{ij}(ul))$

with:

$WARV_{ij}$ = WARV for the r_{ij} restraint; wk = weight of the k structure of the ensemble; $\sum_k w_k = 1$;

$r_{ij}(avg) = (\sum_k w_k\, r_{ij}(k)^{-6})^{-1/6}$; $r_{ij}(ll) = r_{ij}$ restraint lower limit ; $< r_{ij}(ll) = r_{ij}$ restraint upper limit.

References

1. Case, D.A.; Cerutti, D.S.; Cheatham, T.E.I.; Darden, T.A.; Duke, R.E.; Giese, T.J.; Gohlke, H.; Goetz, A.W.; Greene, D.; Homeyer, N.; et al. AMBER 2017.
2. Wang, J.; Wolf, R.M.; Caldwell, J.W.; Kollman, P.A.; Case, D.A. Development and testing of a general amber force field. *J. Comput. Chem.* **2004**, *25*, 1157–1174.
3. Gordon, M.S.; Schmidt, M.W. Advances in electronic structure theory: GAMESS a decade later. In *Theory and Applications of Computational Chemistry: the first forty years*; Dykstra, C.E., Frenking, G., Kim K.S., Scuseria, G.E., Eds.; Elsevier: Amsterdam, 2005; pp. 1167–1189.
4. Pastor, R.W.; Brooks, B.R.; Szabo, A. An analysis of the accuracy of Langevin and molecular dynamics algorithms. *Mol. Phys.* **1988**, *65*, 1409–1419.
5. Ryckaert, J.-P.; Ciccotti, G.; Berendsen, H.J.. Numerical integration of the cartesian equations of motion of a system with constraints: molecular dynamics of n-alkanes. *J. Comput. Phys.* **1977**, *23*, 327–341.
6. Sigalov, G.; Scheffel, P.; Onufriev, A. Incorporating variable dielectric environments into the generalized Born model. *J. Chem. Phys.* **2005**, *122*, 094511.
7. Sigalov, G.; Fenley, A.; Onufriev, A. Analytical electrostatics for biomolecules: Beyond the generalized Born approximation. *J. Chem. Phys.* **2006**, *124*, 124902.
8. Mongan, J.; Simmerling, C.; McCammon, J.A.; Case, D.A.; Onufriev, A. Generalized Born Model with a Simple, Robust Molecular Volume Correction. *J. Chem. Theory Comput.* **2007**, *3*, 156–169.
9. Kelley, L.A.; Gardner, S.P.; Sutcliffe, M.J. An automated approach for clustering an ensemble of NMR- derived protein structures into conformationally related subfamilies. *Protein Eng.* **1996**, *9*, 1063–1065
